# Inclusion of hypocretin-1 improved performance of poor sleep quality prediction for elderly patients with acute ischemic stroke: a prospective cohort study

**DOI:** 10.3389/fnagi.2024.1509846

**Published:** 2025-01-07

**Authors:** Ting Fu, Weiwei Zhang, Rongrong Guo, Shuang He, Saiying Yu, Huiying Wang, Yunfeng Zhang, Ying Wu

**Affiliations:** ^1^School of Nursing, Capital Medical University, Beijing, China; ^2^Department of Laboratory Medicine, Affiliated Hospital of Nantong University, Nantong, Jiangsu, China; ^3^Department of Stroke Center, Affiliated Hospital of Nantong University, Nantong, Jiangsu, China; ^4^Department of Neurology, Affiliated Hospital of Nantong University, Nantong, Jiangsu, China

**Keywords:** Hypocretin-1, sleep quality, depression, acute ischemic stroke, elderly

## Abstract

**Background:**

Hypocretin-1 is a vital neurotransmitter in regulating the sleep–wake cycle and provides neuroprotection against cerebral ischemia. We aims to develop a poor sleep quality predictive model for elderly population with acute ischemic stroke.

**Methods:**

A total of 183 consecutively elderly patients were included in the prospective cohort study. Sleep quality was assessed by the Pittsburgh Sleep Quality Index (PSQI). Cerebrospinal fluid samples were taken within 24 h of acute ischemic stroke onset. After selecting optimal predictors via univariate analysis and bootstrapped stepwise logistic regression, the predictive model was developed. The models were internally validated and evaluated comprehensively via discrimination, calibration, and clinical utility.

**Results:**

The prevalence of poor sleep (PSQI >7) was 64.5% among elderly individuals experiencing acute ischemic stroke. The study developed a predictive model using hypocretin-1, hypertension, stroke history, the National Institutes of Health Stroke score, and depression. Adding hypocretin-1 (as continuous variable) significantly improved the model performance greatly, as the area under the receiver operating characteristic curve increased from 0.799 to 0.845 (*p* < 0.001). The optimal cutoff value for hypocretin-1 was 74.94 pg/mL. Adding hypocretin-1 (as binary variable) significantly improved the model performance greatly, as the AUC increased from 0.799 to 0.857 (*p* < 0.001).

**Conclusion:**

Reduced cerebrospinal fluid levels of hypocretin-1 at admission were an independent poor sleep quality predictor and the model demonstrated superior performance. The combination of hypocretin-1 could offer valuable prognostic information for post-stroke sleep quality in elderly patients with acute ischemic stroke.

## 1 Introduction

Stroke is the third most common cause of death worldwide, and it is the leading cause of death in China, with the country accounting for approximately one-third of all stroke-related deaths (Wang et al., 2022). Ischemic stroke, which make up 60–80% of these cases, is projected to double every 10 years due to the aging of population, which will pose a significant health threat for patients (Benjamin et al., 2018; Wang et al., 2017). As the ongoing improvements in treatment level and nursing technology, the survival rate of stroke has been gradually increased. By 2050, the number of stroke survivors will reach 200 million worldwide (Brainin et al., 2020). Most elderly survivors endure various complications, including sleep disorder, post-stroke depression (PSD), and disability, which results in the impairments of health-related quality of life (Khot and Morgenstern, 2019; Yousufuddin and Young, 2019; Li et al., 2019).

Poor sleep is one of the major prevalent and burdening symptoms and can have debilitating effect on physical and/or mental function and health (Davis et al., 2019). Elderly patients of acute ischemic stroke often face multiple sleep issues, including poor sleep quality, difficulty initiating asleep, nocturnal awakenings, and excessive daytime sleeping (Fan et al., 2022). A meta-analysis found that 49–66% of stroke patients suffered from poor sleep quality, and more than half were older than 60 years old (Luo et al., 2023). Sleep disturbances are strongly correlated with increased risk for emotional disorders, cognitive impairment, and neurodegenerative diseases in older adults (Culebras, 2021). A reliable predictive model for poor sleep quality is essential to pinpoint high-risk patients early and formulate appropriate preventive strategies timely.

Previous studies have also reported that demographic information, lifestyle behaviors, clinical characteristics, and laboratory parameters were connected with sleep quality (Pace et al., 2015; Lindley, 2018). A large population-based study showed that neck circumference, body mass index (BMI), waist circumference, age, and the National Institutes of Health Stroke score were the most influential variables in predicting post-stroke sleep-disordered breathing (Brown et al., 2020). While, Brown et al. (2020) suggested that objective tests are needed to differentiate ischemic stroke patients with and without sleep disturbance. Emerging evidence suggests that incorporating a set of biomarkers might improve the sensitivity and specificity for detecting sleep–wake disturbances (Sauchelli et al., 2016).

Hypocretin-1 (Hcrt-1, also known as orexin-A), primarily produced by the lateral hypothalamic neurons, critically regulates the sleep–wake cycle (De Luca et al., 2022). Meanwhile, Hcrt-1 provides neuroprotection against cerebral ischemia–reperfusion injury, reducing neurological deficits and infarct size (Pace et al., 2015). Intriguingly, mean cerebrospinal fluid (CSF) Hcrt-1 concentrations were significantly lower among ischaemic stroke patients compared with control subjects (Kotan et al., 2013). A research based on rat models of ischemic stroke also demonstrated that Hcrt was lowly expressed in brain of rats with ischemic stroke (Wu et al., 2022). Studies in neurodegenerative diseases show an association between poor sleep quality/sleep fragmentation and elevated Hcrt-1 levels in CSF (Liguori et al., 2016; Liguori et al., 2019). Conversely, Friedman et al. (2007) found a negative association between Hcrt-1 concentrations and sleep disturbances. Sauchelli et al. (2016) demonstrated that Hcrt-1 levels significantly correlate with poor sleep quality in patients with anorexia nervosa. However, other research reported no significant link between decreased CSF Hcrt-1 levels and clinical sleep disturbances (Ogawa et al., 2022; Compta et al., 2009). Thus, the relationship between CSF Hcrt-1 levels and sleep remains inconclusive, and evidence on the role of Hcrt-1 on sleep regulation among acute ischemic stroke patients is lack. Whether the Hcrt-1 levels in the CSF might be a useful biomarker for predicting poor sleep quality among elderly acute ischemic stroke patients?

Given the above, the implications of CSF Hcrt-1 levels and their significance as an independent predictor of poor sleep quality in elderly patients with acute ischemic stroke remain unexplored. This study aims to: (1) assess the association between CSF Hcrt-1 levels and sleep quality in elderly acute ischemic stroke patients; and (2) develop a poor sleep quality predictive model and evaluate it is efficacy, with and without the inclusion of Hcrt-1 levels.

## 2 Materials and methods

### 2.1 Study participants

We conducted a prospective cohort study at the Affiliated Hospital of Nantong University from November 2022 to August 2024. Patients with acute ischemic stroke, diagnosed by computed tomography or magnetic resonance imaging reports within 72 h of hospitalization, were consecutively enrolled if they were admitted to the hospital within 7 days after stroke onset. Exclusion criteria were: (1) patients were diagnosed with transient ischemic attacks; (2) patients were younger than 60 years; (3) patients had sleep disorders or the Pittsburgh Sleep Quality Index (PSQI) ≤ 7 score or cannot be obtained through various attempts (asking elderly patients, their family members or main caregivers); (4) patients had a history of central nervous system diseases (except for stroke history); (5) patients had psychiatric disorders; (6) patients suffered from severe conditions like trauma or major organ failure; (7) we missed the data Hcrt-1; (8) patients had irregular sleep schedules due to shift work; (9) patients had communication barriers such as severe hearing loss or dementia; (10) patients were lost to follow up. The study received approval from the Ethics Committee of the Affiliated Hospital of Nantong University (2021-Q094-01), and written informed consent was obtained from all participants or their surrogates upon enrollment.

### 2.2 CSF samples collection and Hcrt-1 measurement

Existing data showed the possible interaction between Hcrt-1 and the circadian system (Appelbaum et al., 2010). The levels of hypothalamic Hcrt-1 were higher during the daily active period than at night (Yoshida et al., 2001). To avoid the fluctuation feature of Hcrt-1 levels, all CSF samples were taken from 8:00 am to 9:00 am after overnight fasting within 1 day of acute ischemic stroke onset. CSF samples were collected via lumbar puncture using atraumatic needles in polypropylene tubes. Samples were centrifuged at 400 × g for 10 min at 4°C immediately after collection to remove cells and debris, then aliquots were frozen at −80°C until analysis. Hcrt-1 levels were measured using a sandwich ELISA technique (ml057868, Mlbio, Shanghai, China).

### 2.3 Sleep quality measurement

Sleep quality was assessed using the Chinese version of the PSQI both at admission and 1 month post-stroke (Niu et al., 2023). The PSQI comprises of 19 items across seven components: sleep quality, duration, latency, disturbances, efficiency, medication use, and daytime dysfunction. Items are scored from 0 (“no symptoms”) to 3 (“three or more times per week”), with a total score range of 0–21. Existing evidence shows the variation in the prevalence of poor sleep quality by different cutoff points. In the current study, we choose a cutoff point of 7 scores as the majority of studies used this cutoff in older patients (≥60 years) with stroke (Huang et al., 2022).

### 2.4 Collection of other poor sleep risk factors

According to expert advice and literature, risk factors for poor sleep were categorized as demographic, disease-related, laboratory-related, iatrogenic, and psychological factors (Fatahzadeh and Glick, 2006; Campbell et al., 2019). Demographic characteristics, including age, gender, BMI, residence, marital status, education, annual income, and medical insurance were obtained during the first 24 h after admission. Disease-related factors (involving vascular risk factors, laboratory parameters, disease severity, and functional status), iatrogenic factors (including mechanical ventilation, physical restraint, gastric tube, and indwelling catheter), and laboratory parameters were collected during the first 24 h after admission. Disease severity and functional status were assessed by the NIH Stroke Scale (NIHSS) assessed by the Barthel Index (BI).

The Chinese version of the Generalized Anxiety Disorder (GAD-7) questionnaire and the Patient Health Questionnaire (PHQ-9) were used to measure levels of anxiety and depression within 24 h after admission, respectively (Dajpratham et al., 2020). The GAD-7 and PHQ-9 have both been shown to be capable of screening for their respective conditions and are valid measures of clinically diagnosed anxiety and depression. Scores of <5 represent “no anxiety” or “no depression” and scores of ≥5 represent the presence of anxiety or depression for each measure (Fan et al., 2022).

### 2.5 Data collection

Data were collected from enrolled participants and their relatives by using paper-based questionnaires, administered by the same physician and entered into a computer database by two research assistants. All results were double-checked against the original data before analysis.

### 2.6 Sample size

The sample size was calculated using PASS software (version 21.0), aiming for a clinically meaningful 5% improvement in the area under the receiver operating characteristic curve (AUC) of the poor sleep quality predictive model by including Hcrt-1. Based on a target AUC of 0.775 and a poor sleep quality prevalence of 40.2%, we aimed for 80% power and a 0.05 alpha level, resulting in a minimum sample size of 176 for this cohort (Huang et al., 2022; Geng et al., 2023).

### 2.7 Statistical analysis

To avoid the reduction in statistical efficiency and bias caused by eliminating participants with incomplete data directly, we used Multiple Imputation by Chained Equations, running 10 iterations per imputation. Continuous variables were presented as means ± standard deviation or medians (interquartile range) and analyzed using *t*-tests, ANOVA, or Mann–Whitney *U* tests. For the variables with a two-tailed *p-value* < 0.10 in the univariate analysis, we carried out a bootstrapped forward stepwise logistic regression to eliminate the interference of confounding factors on the results and determine the optimal predictors. Optimal Hcrt-1 cutoff points were determined by receiver operating characteristic (ROC) curve analysis to optimize sensitivity and specificity. Two models were developed: Model 1 (without Hcrt-1) and Model 2 (including Hcrt-1 as a continuous variable and binary variable, separately). Model performance was over-fit using 1,000 bootstrap samples and evaluated through ROC curves, calibration plots, and decision curve analysis. We performed several sensitivity analyses to verify model stability, including restricting the analyses to elderly individuals without missing predictors and analyses excluding patients with physical restraint or mechanical ventilation to control for confounding biases. Statistical analyses were conducted using SPSS (version 26.0), R Studio (version 4.2.0), and GraphPad Prism (version 8.0).

## 3 Results

### 3.1 Baseline characteristics of the patients according to sleep quality

**Figure 1:**
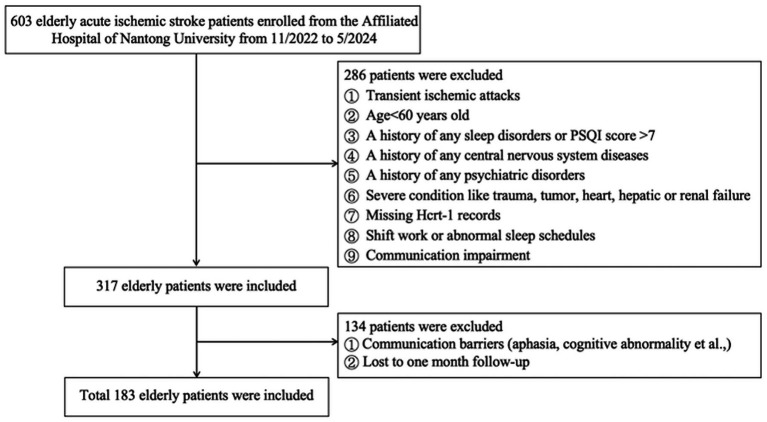
Study flow diagram.

Initially, 603 elderly patients with acute ischemic stroke were enrolled. Among them, 286 were exclude after using the exclusive criteria and 134 individuals lost to follow-up. As shown in Figure 1, a total of 183 patients were included for final analysis. Among them, 65.03% were female and the median (IQR) age was 74.00 (66.80, 81.10) years.

### 3.2 Comparison of the characteristics between good and poor sleepers in elderly patients with acute ischemic stroke

**Figure 2:**
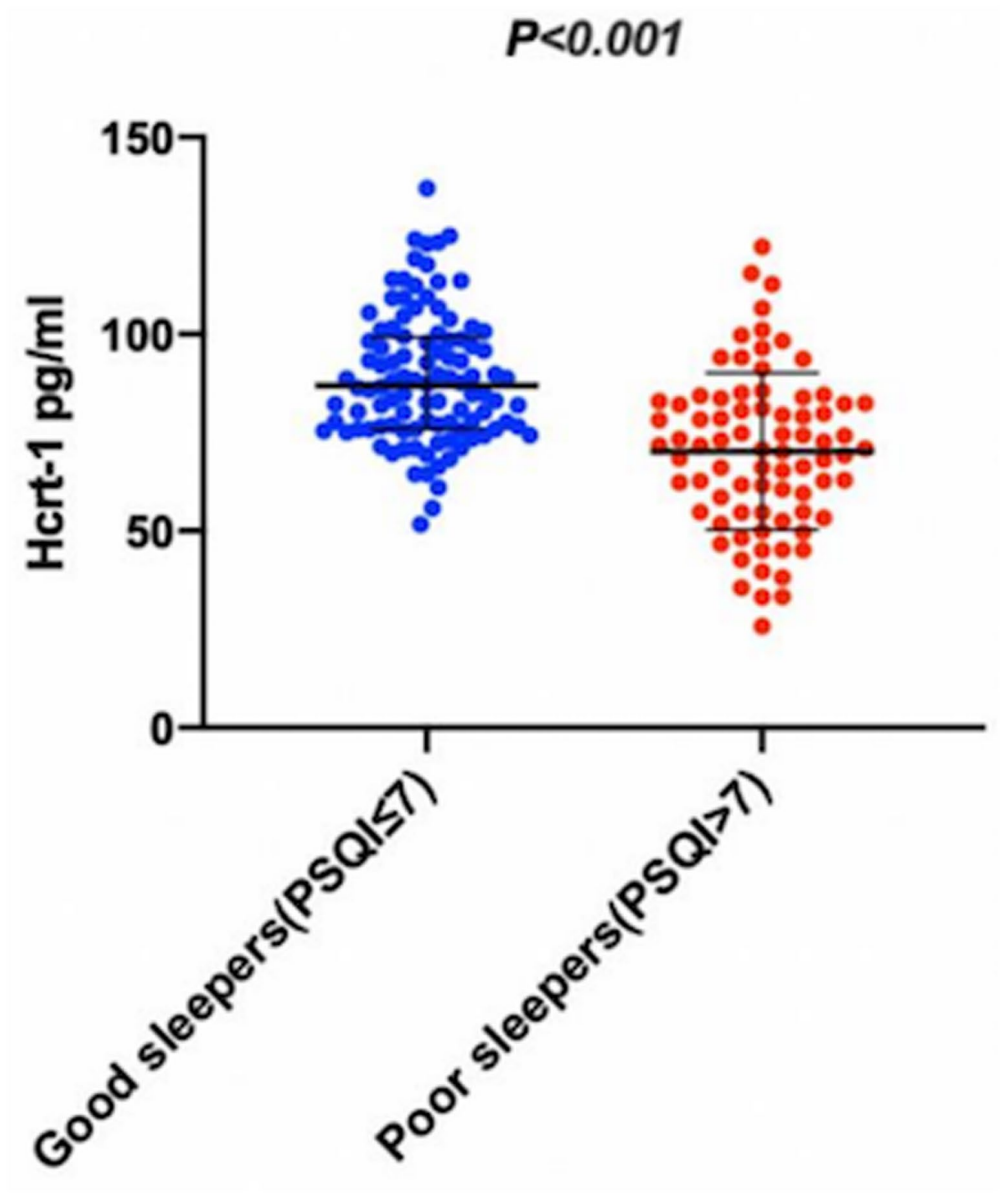
The CSF Hcrt-1 levels between elderly acute ischemic stroke patients with poor sleep quality and good sleep quality. The CSF Hcrt-1 levels between good sleepers and poor sleepers. The horizontal lines indicate median levels and interquartile ranges (IQRs). *p*-values refer to Mann–Whitney Utests for differences between groups. Hcrt-1: hypocretin-1.

The missing rates for variables are detailed in Supplementary Table S1. After addressing these missing data, the characteristics of the participants were summarized in Table 1, which shows baseline characteristics. Comparison of poor sleepers and good sleepers was summarized in Table 1. The data showed that 79 (43.17%) patients had poor sleep quality (PSQI>7) at the one-month follow-up. Poor sleepers had significantly higher NIHSS scores, increased uric acid levels, more depressive and more anxious, with a tendency to lower functional status (BI) than that of good sleepers (*p* < 0.05). Additionally, CSF Hcrt-1 levels were significantly lower in poor sleepers [70.94 pg/mL (IQR, 54.82–82.40)] than in good sleepers [86.91 pg/mL (IQR, 76.16–99.05)] (*p* < 0.001) as illustrated in Figure 2. The optimal cutoff value for Hcrt-1 was 74.94 pg/mL. Supplementary Table 1 showed that the number of low Hcrt-1 levels (≤74.94 pg/mL) in poor sleepers was higher than in good sleepers (18.30% vs 62.00%, *p* < 0.001).

**Table 1:** Comparison between poor and good sleepers in older patients with acute ischemic stroke.

	PSQI ≤ 7 (*n* = 104)	PSQI > 7(*n* = 79)	*p*
Gender, female^b^	64 (61.54)	53 (67.09)	0.439
Age (years)^c^	73.00 (66.50, 79.00)	75.00 (67.00, 82.00)	0.164
BMI (kg/m^2^)^a^	24.38 ± 3.37	23.98 ± 4.39	0.502
Residence^b^			0.857
City	46 (44.23)	36 (45.57)	
Country	58 (55.77)	43 (54.43)	
Marital status^b^			0.102
Married	83 (79.81)	51 (64.56)	
Unmarried	2 (1.92)	1 (1.27)	
Widowed	17 (16.35)	25 (31.65)	
Divorced	2 (1.92)	2 (2.53)	
Education^b^			0.493
Primary school and below	34 (32.69)	23 (29.11)	
Junior high school or technical secondary school	28 (26.92)	27 (34.18)	
High school or junior college	34 (32.69)	23 (29.11)	
Bachelor degree or above	16 (15.38)	15 (18.99)	
Income/year (RMB)^b^			0.615
<15,000	42 (40.38)	27 (34.18)	
15,000–33,000	35 (33.65)	27 (34.18)	
>33,000	27 (25.96)	25 (31.65)	
Medical insurance, yes^b^	88 (84.62)	66 (83.54)	0.898
Current Drinking, yes^b^	21 (20.19)	23 (29.11)	0.162
Current smoking, yes^b^	19 (18.27)	23 (29.11)	0.084
Stroke history, yes^b^	12 (11.54)	19 (24.05)	***0.025***
Comorbidities^b^			
Hypertension	55 (52.88)	57 (72.15)	***0.008***
DM	41 (39.42)	32 (40.51)	0.882
Hyperlipidemia	51 (49.04)	35 (44.30)	0.525
Medications use^b^			
Antiplatelet agents	5 (4.81)	3 (3.80)	0.741
Anticoagulant	0 (0.00)	1 (1.27)	0.432
Lipid-lowering agents	2 (1.92)	3 (3.80)	0.441
Lesion location^b^			0.663
Frontal lobe	16 (15.20)	11 (14.30)	
Parietal lobe	11 (10.90)	9 (10.80)	
Temporal lobe	7 (6.50)	7 (8.86)	
Occipital lobe	4 (4.30)	5 (6.33)	
Basal ganglia	43 (41.30)	34 (43.04)	
Cerebellum	9 (8.70)	4 (5.06)	
Brainstem	14 (13.00)	9 (10.80)	
Lmphocyte count (10^9^/L)^c^	1.33 (1.03, 1.76)	1.24 (0.81, 1.60)	0.229
Neutrophil count (10^9^/L)^c^	5.02 (3.88, 7.04)	5.10 (3.64, 7.27)	0.245
WBC count (10^9^/L)^c^	7.13 (5.77, 9.52)	7.05 (5.35, 9.45)	0.605
Hb (g/L)^a^	125.23 ± 23.51	120.41 ± 24.77	0.181
PLT (10^9^/L)^c^	185.00 (129.00, 138.00)	170.00 (138.00, 231.00)	0.512
UA (μmol/L)^c^	353.00 (313.00, 418.50)	286.00 (396.50, 508.00)	**0.023**
TB (μmol/L)^c^	13.13 (10.66, 19.19)	13.08 (9.35, 17.30)	0.404
TG (μmol/L)^c^	1.25 (0.96, 2.00)	1.22 (0.86, 1.71)	0.424
TC (μmol/L)^c^	4.02 (3.37, 4.69)	3.45 (2.83, 4.26)	0.360
DB (μmol/L)^c^	2.59 (1.79, 4.05)	2.70 (1.66, 4.63)	0.238
LDL (mmol/L)^c^	2.37 (1.88, 2.83)	2.11 (1.68, 2.79)	0.438
HDL (mmol/L)^c^	0.99 (0.87, 1.23)	1.00 (0.79, 1.16)	0.283
ALT (IU/L)^c^	23.00 (15.50, 40.00)	18.50 (13.00, 34.00)	0.546
AST (IU/L)^a^	69.78 ± 112.02	52.63 ± 84.55	0.257
Serum albumin (mmol/L)^c^	139.20 (137.25, 141.05)	139.10 (137.00, 141.10)	0.756
Serum potassium (mmol/L)^c^	4.00 (3.80, 4.20)	4.20 (673.80, 4.50)	0.415
Serum calcium (mmol/L)^c^	2.24 (2.17, 2.29)	2.20 (2.09, 2.30)	0.626
CRP (mg/L)^c^	4.06 (1.44, 12.24)	7.83 (2.79, 21.76)	0.278
CSF IgG (mg/dl)	8.96 (2.93, 25.70)	4.56 (2.93, 25.06)	0.748
CSF IgG Index	0.51 (0.46, 0.52)	0.55 (0.45, 0.57)	0.190
CSF albumin (mg/dl)	66.50 (18.00, 117.00)	20.10 (18.00, 117.00)	0.659
CSF 24 h intrathecal synthesis rate	−0.40 (−3.82, 7.33)	−0.40 (−3.91, 7.62)	0.903
NIHSS score^c^	3 (3, 4)	4 (3, 5)	**<0.001**
BI ^a^	61.39 ± 21.34	57.41 ± 21.27	0.248
Mechanical ventilation^b^	7 (6.73)	8 (10.13)	0.428
Physical restraint^b^	13 (12.50)	17 (21.52)	0.111
Gastric tube^b^	2 (1.92)	6 (7.59)	0.063
Indwelling catheter^b^	18 (17.31)	19 (24.05)	0.271
Depression (PHQ-9 ≥ 5 score)^b^	21 (20.19)	48 (60.76)	**<0.001**
Anxiety (GAD-7 ≥ 5 score)^b^	12 (11.54)	18 (22.78)	**0.042**
Hcrt-1 ≤ 74.94 pg/mL^b^	19 (18.27)	49 (62.03)	**<0.001**

^a^Means ± SD, ^b^number (percentages), ^c^median (25% percentile, 75% percentile). PSQI, Pittsburgh Sleep Quality Index; BMI, body mass index; DM, diabetes; WBC, white blood cell; Hb, hemoglobin; PLT, platelet; UA, Uric acid; TC, total cholesterol; TG, triglyceride; TB, total bilirubin; DB, direct bilirubin; HDL, high density lipoprotein; LDL, low density lipoprotein; ALT, alanine aminotransferase; AST, aspartate aminotransferase; CRP, C-reactive protein; NIHSS, National Institutes of Health Stroke Scale; BI, Barthel Index; PHQ-9, patient health questionnaire-9; GAD-7, generalized anxiety disorder-7. Hcrt-1, hypocretin-1. The bold value means *p* < 0.05 and it has statistical significance.

### 3.3 Model development

**Figure 3:**
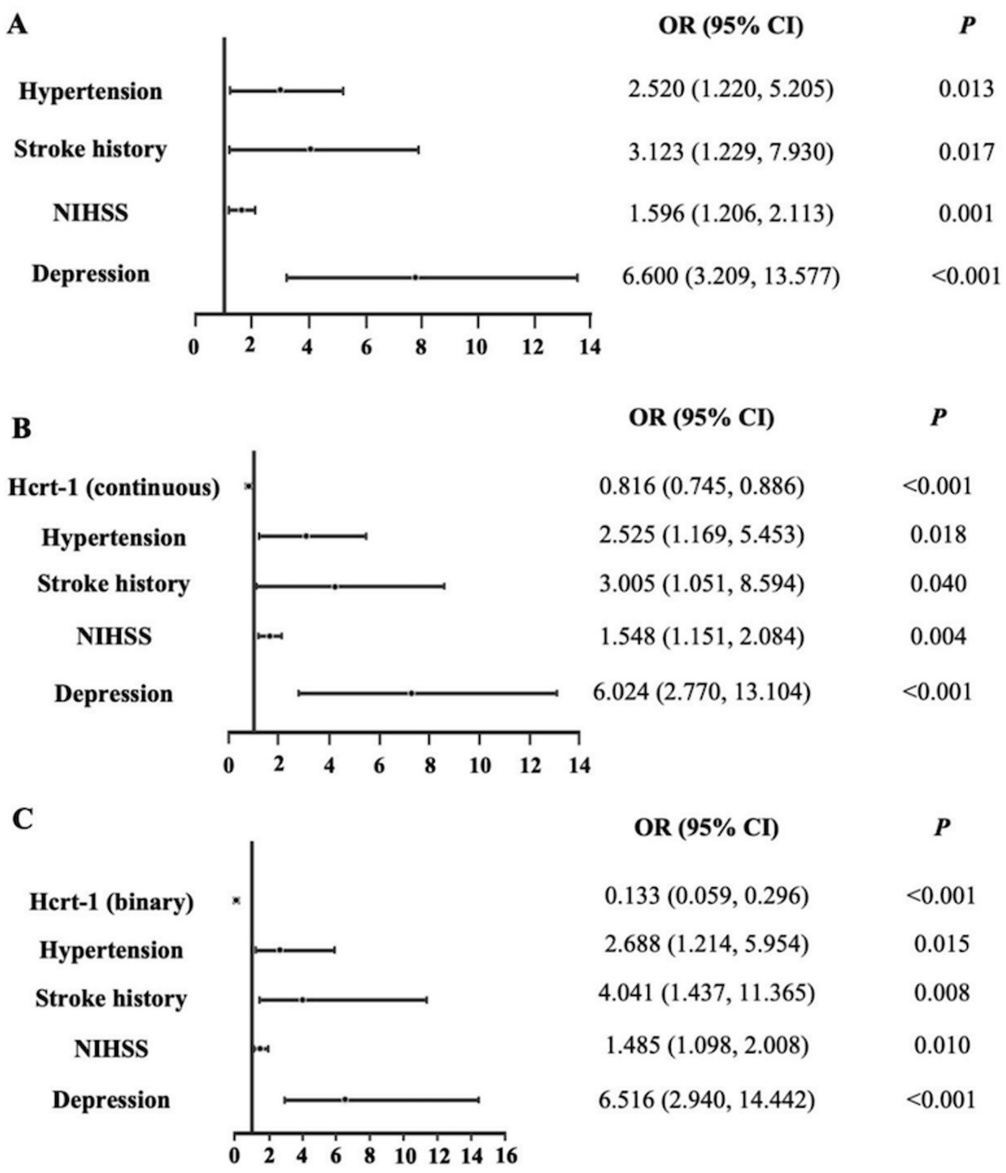
Logistic regression models for the complete dataset. **(A)** Logistic regression of Model 1 for the complete dataset. **(B)** Logistic regression of Model 2 (Hcrt-1 as continuous variable) for the complete dataset. **(C)** Logistic regression of Model 2 (Hcrt-1 as binary variable) for the complete dataset.

Using bootstrapped forward stepwise logistic regression, four variables were identified as independent predictors of poor sleep quality in Model 1 (Figure 3A). These included hypertension, stroke history, NIHSS score, and depression. Incorporating Hcrt-1 levels (as continuous variable) into Model 1 and rerunning logistic regression created Model 2 (Figure 3B). Based on the smaller Akaike’s Information Criterion values (Model 1: 204.88; Model 2: 176.78), Bayesian Information Criterion values (Model 1: 220.93; Model 2: 204.19), and likelihood tests (Likelihood Ratio: 30.10, *p* < 0.001), Model 2 was selected as the final predictive model for poor sleep quality. Hcrt-1 was also treated as a binary variable to test model 2 (Figure 3C). Based on the smaller Akaike’s Information Criterion values (Model 1: 201.13; Model 2: 179.46), Bayesian Information Criterion values (Model 1: 220.12; Model 2: 198.72), and likelihood tests (Likelihood Ratio: 27.43, *p* < 0.001), Model 2 was selected as the final predictive model for poor sleep quality.

### 3.4 Model evaluation and the role of Hcrt-1

**Figure 4:**
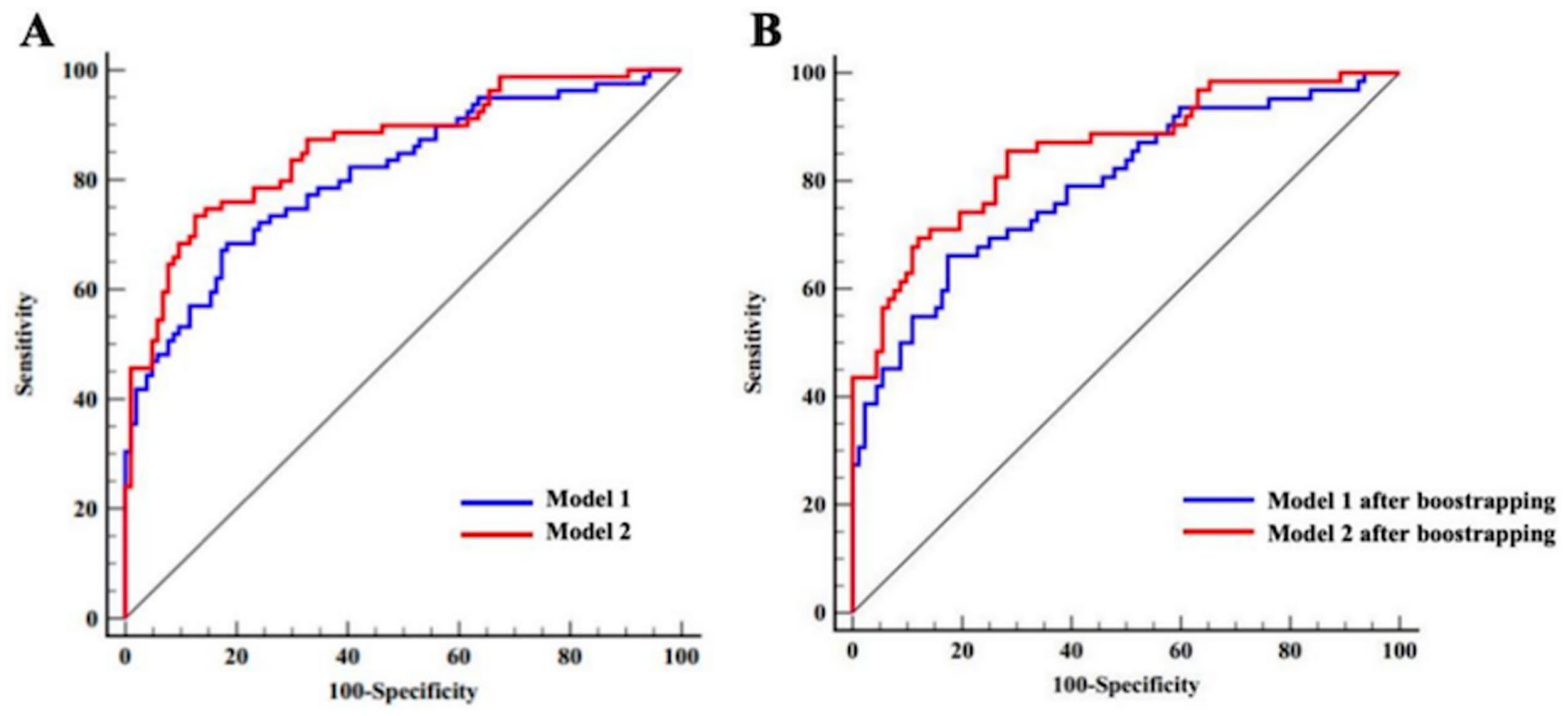
ROC curves of Model 1 and Model 2 for the complete dataset (Hert-1 as continuous variable). **(A)** ROC curves of Model 1 and Model 2 prior to bootstrapping. **(B)** ROC curves of Model 1 and Model 2 after bootstrapping.

Before internal validation, the AUCs for Model 1 and Model 2 (Hcrt-1 as continuous variable) were 0.809 (95% CI: 0.745–0.873) and 0.860 (95% CI: 0.805–0.915), respectively. Post-bootstrapping, the AUCs remained robust at 0.799 (95% CI: 0.733–0.864) for Model 1 and 0.845 (95% CI: 0.788–0.902) for Model 2. The Delong test confirmed the superior discrimination of Model 2, which showed an increase in AUC of 5.1% (*p* < 0.001) and 4.6% (*p* < 0.001) in the initial cohort and after bootstrap internal validation, respectively (Figure 4).

**Figure 5:**
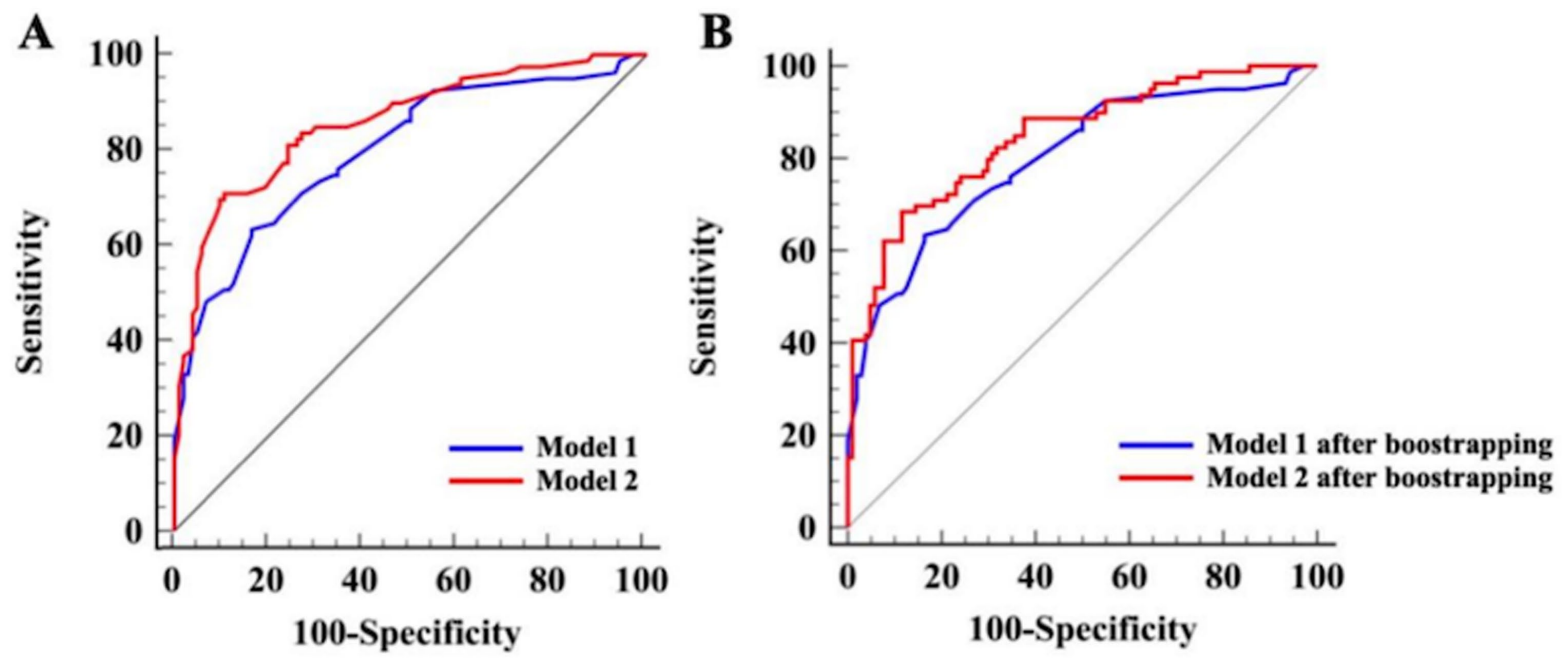
ROC curves of Model 1 and Model 2 for the complete dataset (Hcrt-1 as binary variable). **(A)** ROC curves of Model 1 and Model 2 prior to bootstrapping. **(B)** ROC curves of Model 1 and Model 2 after bootstrapping.

The same model evaluation and Hcrt-1 role treated as binary variable were conducted. Before internal validation, the AUCs for Model 1 and Model 2 (Hcrt-1 as binary variable) were 0.809 (95% CI: 0.745–0.873) and 0.872 (95% CI: 0.816–0.921), respectively. Post-bootstrapping, the AUCs remained robust at 0.799 (95% CI: 0.733–0.864) for Model 1 and 0.857 (95% CI: 0.802–0.913) for Model 2. The Delong test confirmed the superior discrimination of Model 2, which showed an increase in AUC of 6.5% (*p* < 0.001) and 5.8% (*p* < 0.001) in the initial cohort and after bootstrap internal validation, respectively (Figure 5).

**Figure 6:**
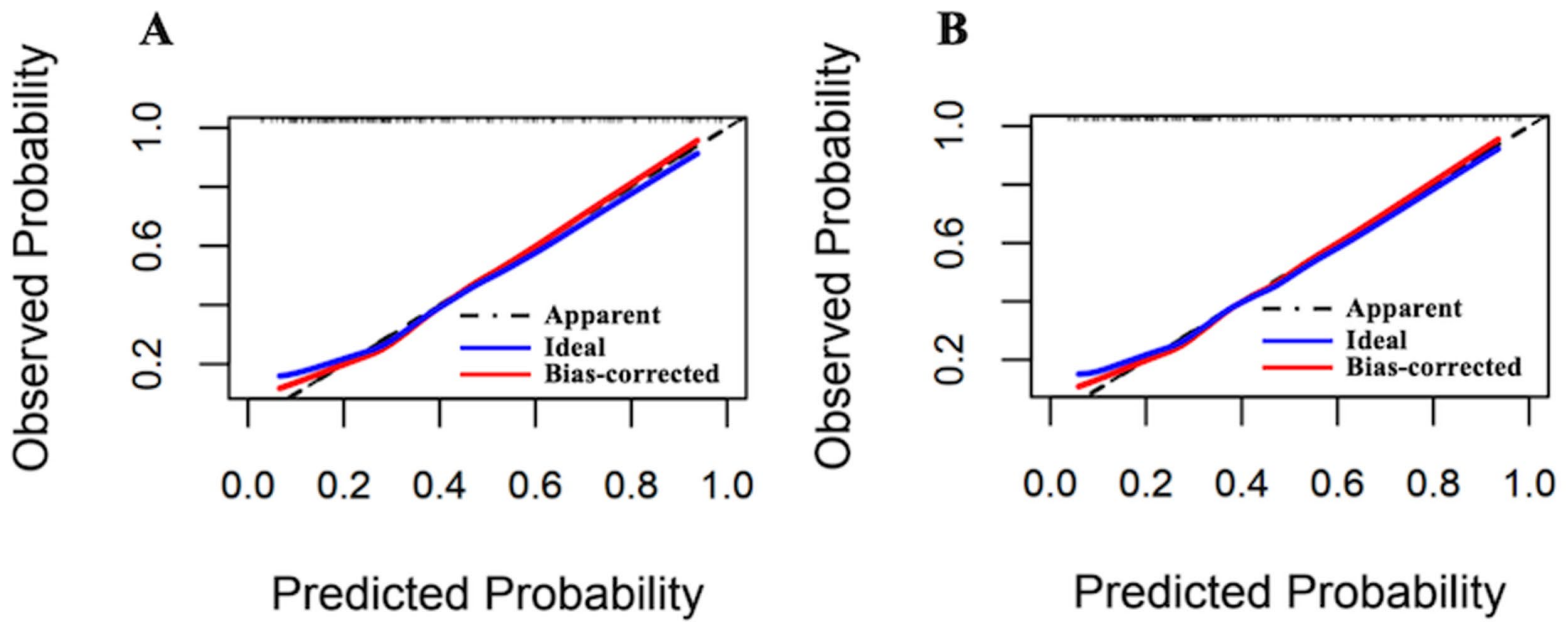
Calibration plots of Model 1 and Model 2 for the complete dataset (Hcrt-1 as continuous variable). **(A)** Calibration plots of Model 1 after bootstrapping for the complete dataset. **(B)** Calibration plots of Model 2 after bootstrapping for the complete dataset.

**Figure 7:**
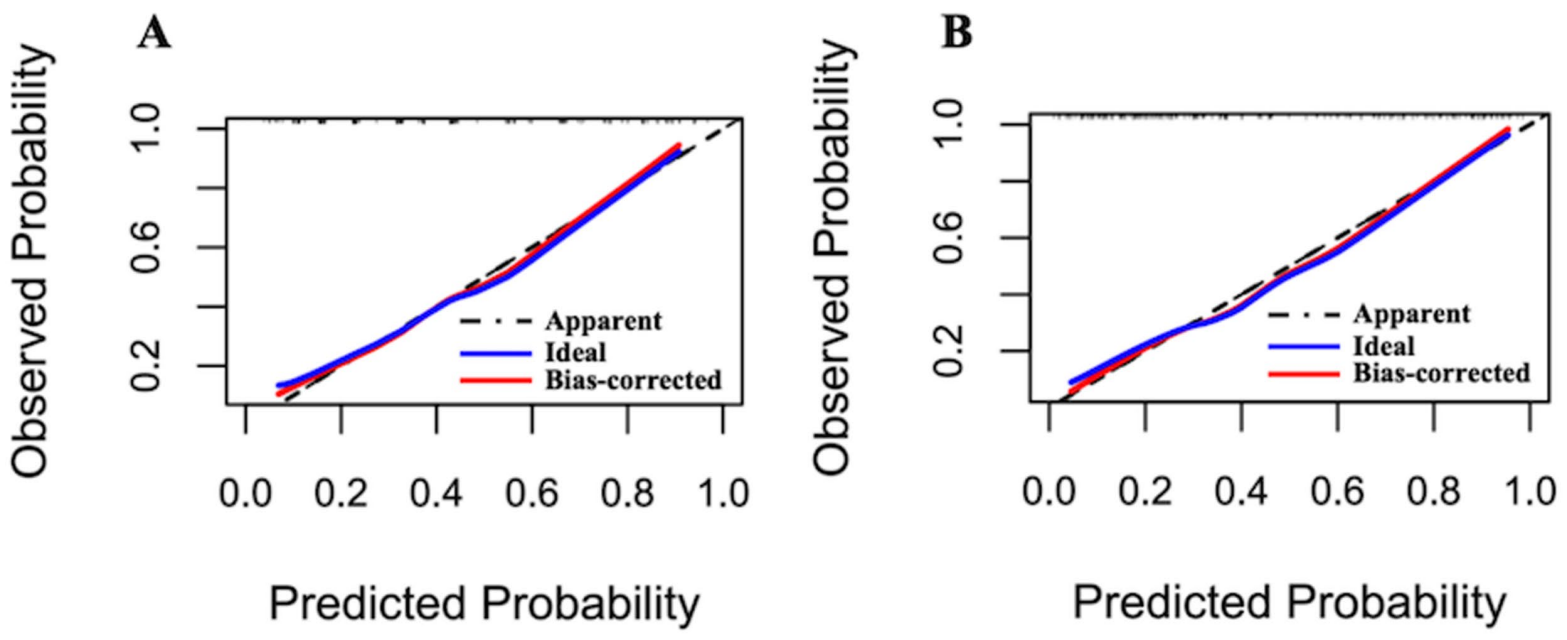
Calibration plots of Model 1 and Model 2 for the complete dataset (Hcrt-1 as binary variable). **(A)** Calibration plots of Model 1 after bootstrapping for the complete dataset. **(B)** Calibration plots of Model 2 after bootstrapping for the complete dataset.

**Figure 8:**
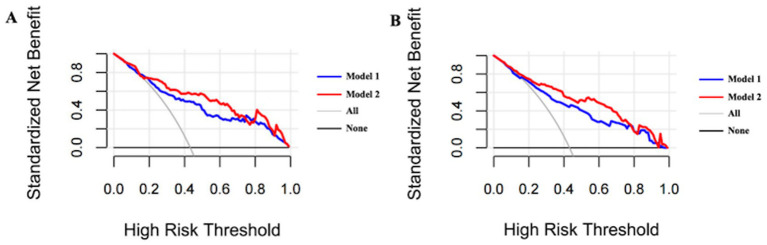
Bias-corrected decision curve analysis of Model 1 and Model 2 for the complete dataset. **(A)** Bias-corrected decision curve analysis of Model 1 and Model 2 after bootstrapping in current cohort (Hcrt-1 as continuous variable). **(B)** Bias-corrected decision curve analysis of Model 1 and Model 2 after bootstrapping in current cohort (Hcrt-1 as binary variable).

Following optimism-adjusted bootstrap validation, both models demonstrated good calibration as shown in the calibration plot (Figures 6, 7). The decision curve analysis (Figure 8) indicated that Model 2 offered superior clinical utility across most threshold probabilities, except at the extreme ranges of 0.9–1.0.

### 3.5 Sensitivity analyses

The variable selection and model performance results from the sensitivity analyses were consistent with those obtained from the complete dataset, as detailed in Supplementary material (p. 6–17).

## 4 Discussion

Our study showed that 43.17% patients after acute ischemic stroke developed poor sleep quality at 1 month. Low CSF Hcrt-1 levels (≤74.94 pg/mL) were significantly associated with poor sleep quality. In addition, we developed the predictive model, including five predictors (hypertension, stroke history, NIHSS, depression, and Hcrt-1), and demonstrated the Hcrt-1 levels as a significant predictor to improve the clinical performance of the poor sleep quality predictive model for elderly patients with acute ischemic stroke greatly. It indicates that identified risk factors have a good predictive performance for the development of poor sleep quality. To the best of our knowledge, this is the first poor sleep quality predictive model incorporating CSF Hcrt-1 as an independent predictor among elderly patients with acute ischemic stroke.

Most research focuses on pre-stroke sleep disturbance and post-stroke depression (Fan et al., 2022; Dong et al., 2021). However, little is known about the role of depression in post-stroke poor sleep quality. It is well known that sleep disturbance have been considered the core secondary symptom of depression in the past decades. Fortunately, depression has been usually regarded as a risk factor for developing sleep disturbance (Fang et al., 2019). One meta-analysis study has suggested that sleep disturbance and depression had certain common physiopathology, including the activation of neurotransmitter and neuroendocrine systems like serotonin, oxidative stress, and inflammatory response (Wang et al., 2015). The possible hypothesis may be that levels of serotonin, norepinephrine and dopamine metabolites are decreased, and abnormal genetic regulation of serotonergic transmission has been observed in depression (Krishnan and Nestler, 2008). Those changes disturb the function of cholinergic and monoaminergic neurons, which also play a vital role in sleep parameters across all phases of sleep architecture (Wang et al., 2015).

In our study, stroke severity was another risk factor for sleep quality, which was similar to previous studies (Bassetti et al., 2006). A possible explanation is that limb movement disorders are common among acute ischemic stroke patients (Seitz et al., 2011). Some elderly patients develop myotonia and muscle tension which may lead to inconvenient movements, nocturnal urination, and difficulty in coughing up phlegm and getting out of bed, which all affect sleep quality (Luik et al., 2013).

Additionally, a history of stroke was an independent predictor of elderly patients with acute ischemic stroke (Moerch-Rasmussen et al., 2016; Oza et al., 2017). However, Huang et al. (2022) examining sleep disturbance in early acute ischemic stroke patients have found no association between stroke history and sleep quality. One research has shown that telomere length, as an indicator of aging, was significantly associated with stroke recurrence in ischemic stroke patients aged >65 years, but not in younger patients (Moerch-Rasmussen et al., 2016). In the present study, all patients were over 60 years, which may account for the differences in our findings compared to other reported findings (Huang et al., 2022).

Hcrt-1 concentration in the CSF might be a useful biomarker for the assessment of progression of brain tissue damage during the early stages of ischemic stroke (Kotan et al., 2013). Acute ischemic stroke patients with subjective sleep problems had lower CSF Hcrt-1 levels compared to controls without sleep disturbance (Liguori et al., 2016). The relationship between Hcrt-1 and poor sleep quality could be best explained by the following mechanisms. According to recent studies, Hcrt-1 is involved in blood pressure regulation (Abreu et al., 2020; Huber et al., 2018). Hcrt knock-out mice and Hcrt neuron-ablated transgenic rats have lower basal blood pressure (Li et al., 2013). One study reported that the blockage of Hcrt receptors attenuates blood pressure in hypertensive rats (Clifford et al., 2015). Another study demonstrated that intravenous administration of Hcrt decreases infarct volume by increasing cerebral blood flow (Bulbul et al., 2008). Wu et al. (2022) have shown that Hcrt-1 was lowly expressed in the brains of rats with ischemic stroke. Upregulating Hcrt-1 could improve sleep architecture of post-stroke rats, such as total sleep time, non-rapid eye movement frequency, and frequency of rapid eye movement (Wu et al., 2022). Given that hypertension is a risk factor for stroke, the promotion of Hcrt-1 secretion might be involved in the onset of sleep problems by regulating blood pressure (Wang et al., 2011; Gorgui et al., 2014).

Neuroinflammation, involving the over-expression of inflammatory mediators and proinflammatory cytokines, might be another primary hypothesis for the mechanism of poor sleep development and Hcrt-1. Inflammation conditions lead to Hcrt-1 neuron damage and a large increase in the amounts of wakefulness (Gerashchenko and Shiromani, 2004). A recent study reported that acute ischemic stroke people were reported to exhibit abnormal expression of pro-inflammatory cytokines, which might result in an increased risk for poor sleep quality (Song et al., 2015). Tumor necrosis factor alpha (TNF-*α*), an important neuroinflammatory cytokine, impaired the function of the Hcrt-1 system and regulated the process of sleep (Zhan et al., 2011; Zhan et al., 2019).

Several studies highlighted the anti-inflammatory function of Hcrt-1 in neuroinflammation diseases and oxidative stress caused by ischemic stroke (Xiong et al., 2013). Intracerebroventricular administration of Hcrt-1 before ischemia stroke reduces infarct size (Harada, 2014). Under ischemic conditions, Hcrt-1 promotes the survival of primary cortical neurons *in vitro* and alleviates neuronal damage by modulating post-ischemic glucose intolerance *in vivo* (Kitamura et al., 2010). Meanwhile, Hcrt-1 regulates infection-induced inflammation by modulating the IL-6 and TNF-α in microglia and has a protective role against ischemia stress (Xiong et al., 2013). However, exploring if there is a causal relationship between poor sleep and Hcrt-1 and the executive mechanisms is beyond the current study. Further studies are needed to explore the causal relationship between poor sleep and Hcrt-1 and the executive mechanisms.

The strengths of this study include the performance of the poor sleep quality predictive model for elderly patients with acute ischemic stroke. It was greatly improved after including hypocretin-1 as an independent predictor. Additionally, elderly patients with cerebrospinal fluid levels of hypocretin-1 lower than 74.94 pg/mL at admission were more likely to experience poor sleep quality. The findings emphasize the importance of routine Hcrt-1 assessment and further guide the development of strategies to prevent poor sleep quality for elderly patients with acute ischemic stroke. Moreover, the integration of this model into clinical practice can facilitate a more personalized care paradigm, ensuring that healthcare interventions are not merely reactive but anticipatory, thus fostering a holistic approach to stroke rehabilitation. This shift toward precision medicine could lead to significant advancements in patient-centered care, aligning treatment modalities with the unique needs of elderly stroke patients.

Our study also had several limitations. Firstly, as we included relatively small sample sizes in a single Department of the Stroke Center, the findings of this study cannot be generalized to all elderly patients with acute ischemic stroke in our society. Secondly, we just measured sleep quality and depression with self-report questionnaires. Thirdly, CSF Hcrt-1 levels were measured only at admission, and it is necessary to further explore the relationship between dynamic changes in Hcrt-1 levels and the development of sleep quality. Fourthly, as the included participants had minor stroke, this limits the generalizability of the study. Fifthly, it is difficult to obtain CSF from healthy elderly persons, so the results of Hcrt-1 levels among healthy controls was lack. Sixth, we did not perform an external validation of this predictive model by another independent cohort. Nevertheless, a bootstrap resampling procedure was conducted for internal validation by adjusting for model over-fitting, which demonstrated adequate value and clinical utility of Hcrt-1 of this model in predicting poor sleep quality. Further studies with expanded sample sizes, objective depression and sleep measures, large-scale multi-center studies, and a health control arm should be conducted.

## 5 Conclusion

In summary, this prospective cohort study developed a poor sleep quality predictive model incorporating Hcrt-1 among elderly patients with acute ischemic stroke. The predictive model including Hcrt-1 can improve performance and clinical utility in predicting poor sleep quality. The validations also demonstrated the accurate and stable predictive performance of this model. We postulate that the evidence derived from this study is strong enough to support the implementation of routine Hcrt-1 assessment and further guide the development of strategies to prevent poor sleep quality for elderly patients with acute ischemic stroke.

## Data Availability

The original contributions presented in the study are included in the article/Supplementary material, further inquiries can be directed to the corresponding author.
