# Supplementary material for: Inclusion of hypocretin-1 improved performance of poor sleep quality prediction for elderly patients with acute ischemic stroke: a prospective cohort study
Source: Front Aging Neurosci. 2025 Jan 7;16:1509846. doi: 10.3389/fnagi.2024.1509846 (PMC11747788; doi:10.3389/fnagi.2024.1509846)
Supplement: Supplementary file 1 [file Data_Sheet_1.docx]

Supplementary Material

**Contents**

[**Supplementary imputation method 3**](#_Toc954922755)

[**Supplementary results of the complete dataset 4**](#_Toc1241449254)

[Table S1. Missing rates of data prior to application of multiple imputation in the cohort 4](#_Toc86497726)

[**Supplementary results of the sensitivity analysis by excluding participants with missing predictors 6**](#_Toc2068335510)

[Table S2. Comparison of the baseline characteristics of elderly stroke participants between good sleep quality and poor sleep quality groups in the sensitivity analysis by excluding participants with missing predictors 6](#_Toc1197122581)

Table S3. LRs, AICs, BICs, and AUCs of Model 1 and Model 2 in the sensitivity analysis by excluding participants with missing predictors (Hcrt-1 as continuous variable) 7

Table S4. LRs, AICs, BICs, and AUCs of Model 1 and Model 2 in the sensitivity analysis by excluding participants with missing predictors (Hcrt-1 as binary variable) 8

[Fig S1. Bias-corrected calibration plots of Model 1 and Model 2 for after bootstrapping in the sensitivity analysis by excluding participants with missing predictors (Hcrt-1 as continuous variable) 8](#_Toc264930124)

[Fig S2. Bias-corrected calibration plots of Model 1 and Model 2 for after bootstrapping in the sensitivity analysis by excluding participants with missing predictors (Hcrt-1 as binary variable) 9](#_Toc946993837)

[Figure S3. Bias-corrected decision curve analysis of Model 1 and Model 2 after bootstrapping in the sensitivity analysis by excluding participants with missing predictors 9](#_Toc1124110542)

[**Supplementary results of the sensitivity analysis by excluding participants using mechanical ventilation 1**](#_Toc1512236735)**0**

[Table S5. Comparison of the baseline characteristics of elderly stroke participants between good sleep quality and poor sleep quality groups in the sensitivity analysis by excluding participants with mechanical ventilation 10](#_Toc693842900)

[Table S6. LRs, AICs, BICs, and AUCs of Model 1 and Model 2 in the sensitivity analysis by excluding participants with mechanical ventilation (Hcrt-1 as continuous variable) 11](#_Toc581417090)

[Table S7. LRs, AICs, BICs, and AUCs of Model 1 and Model 2 in the sensitivity analysis by excluding participants with mechanical ventilation (Hcrt-1 as binary variable) 12](#_Toc826437780)

[Fig S4. Bias-corrected calibration plots of Model 1 and Model 2 for after bootstrapping in the sensitivity analysis by excluding participants with mechanical ventilation (Hcrt-1 as continuous variable) 12](#_Toc15539664)

[Fig S5. Bias-corrected calibration plots of Model 1 and Model 2 for after bootstrapping in the sensitivity analysis by excluding participants with mechanical ventilation (Hcrt-1 as binary variable) 1](#_Toc1329611561)3

[Figure S6. Bias-corrected decision curve analysis of Model 1 and Model 2 after bootstrapping in the sensitivity analysis by excluding participants with mechanical ventilation 1](#_Toc66675045)3

[**Supplementary results of the sensitivity analysis by excluding participants using physical restraint 1**](#_Toc1768501228)**4**

[Table S8. Comparison of the baseline characteristics of elderly stroke participants between good sleep quality and poor sleep quality groups in the sensitivity analysis by excluding participants with physical restraint 1](#_Toc2026464516)4

[Table S9. LRs, AICs, BICs, and AUCs of Model 1 and Model 2 in the sensitivity analysis by excluding participants with physical restraint (Hcrt-1 as continuous variable) 15](#_Toc581417090)

[Table S10. LRs, AICs, BICs, and AUCs of Model 1 and Model 2 in the sensitivity analysis by excluding participants with physical restraint (Hcrt-1 as binary variable) 16](#_Toc826437780)

[Fig S7. Bias-corrected calibration plots of Model 1 and Model 2 for after bootstrapping in the sensitivity analysis by excluding participants with physical restraint (Hcrt-1 as continuous variable) 1](#_Toc1845962639)6

[Fig S8. Bias-corrected calibration plots of Model 1 and Model 2 for after bootstrapping in the sensitivity analysis by excluding participants with physical restraint (Hcrt-1 as binary variable) 1](#_Toc397825464)7

[Figure S9. Bias-corrected decision curve analysis of Model 1 and Model 2 after bootstrapping in the sensitivity analysis by excluding participants with physical restraint 17](#_Toc1135980337)

**Supplementary imputation method**

For avoiding the reduction in statistical efficiency and bias caused by eliminating participants with incomplete data directly, Multiple Imputation by Chained Equations was performed to create complete dataset assuming that data is lost at random. The number of iterations in each imputation was set at ten. The imputed 10 datasets were then each analyzed and the study results were pooled into the final study result using “with” function of the Multiple Imputation by Chained Equations package. There is no missing value of the categorical baseline variables and outcome variables in the cohort of this study.

The imputed continuous variables and their respective missing rates in the cohort included: body mass index (2.19%), lymphocyte count (1.64%), neutrophil count (2.73%), white blood cell count (2.19%), hemoglobin(1.64%), platelet (2.73), Uric acid (11.48%), total cholesterol (1.64%), triglyceride (2.19%), total bilirubin (1.09%), direct bilirubin (2.73%), high density lipoprotein (6.01%), low density lipoprotein (6.56%), alanine aminotransferase (3.28%), aspartate aminotransferase (4.37%), serum albumin (9.29%), Serum potassium (12.57%), Serum calcium (8.74%), C-reactive protein (4.92%), National Institutes of Health Stroke Scale (3.83%), Barthel Index (4.92%), Patient health questionnaire (6.01%), Generalized anxiety disorder (4.92%).

**Supplementary results of the complete dataset**

**Table S1. Missing rates of data prior to application of multiple imputation in the cohort**

| **V****ariables** | **Missing rate (%), n=183** |
| --- | --- |
| Gender, female ^b^ | 0 (0.00%) |
| Age (years) ^c^ | 0 (0.00%) |
| BMI (kg/m^2^) ^a^ | 4 (2.19%) |
| Residence ^b^ | 0 (0.00%) |
| Marital status ^b^ | 0 (0.00%) |
| Education ^b^ | 0 (0.00%) |
| Income/year (RMB) ^b^ | 0 (0.00%) |
| Medical insurance, yes ^b^ | 0 (0.00%) |
| Current Drinking, yes ^b^ | 0 (0.00%) |
| Current smoking, yes ^b^ | 0 (0.00%) |
| Stroke history, yes ^b^ | 0 (0.00%) |
| Comorbidities ^b^ | 0 (0.00%) |
| Medications use ^b^ | 0 (0.00%) |
| Lmphocyte count (10^9^/L) ^c^ | 3 (1.64%) |
| Neutrophil count (10^9^/L) ^c^ | 5(2.73%) |
| WBC count (10^9^/L) ^c^ | 4 (2.19%) |
| Hb (g/L) ^a^ | 3 (1.64%) |
| PLT (10^9^/L) ^c^ | 5(2.73%) |
| UA (µmol/L) ^c^ | 21 (11.48%) |
| TC (µmol/L) ^c^ | 3 (1.64%) |
| TG (µmol/L) ^c^ | 4 (2.19%) |
| TB (µmol/L) ^c^ | 2(1.09%) |
| DB (µmol/L) ^c^ | 5(2.73) |
| LDL (mmol/L) ^c^ | 12(6.56%) |
| HDL (mmol/L) ^c^ | 11(6.01%) |
| ALT (IU/L) ^c^ | 6 (3.28%) |
| AST (IU/L) ^a^ | 8 (4.37%) |
| Serum albumin (mmol/L) ^c^ | 17 (9.29%) |
| Serum potassium (mmol/L) ^c^ | 23 (12.57%) |
| Serum calcium (mmol/L) ^c^ | 16 (8.74%) |
| CRP (mg/L) ^c^ | 9 (4.92%) |
| NIHSS score ^c^ | 7 (3.83%) |
| BI ^a^ | 3 (1.63%) |
| Mechanical ventilation ^b^ | 0 (0.00%) |
| Physical restraint ^b^ | 0 (0.00%) |
| Gastric tube ^b^ | 0 (0.00%) |
| Indwelling catheter ^b^ | 0 (0.00%) |
| Depression (PHQ-9≥5 score) ^b^ | 11 (6.01%) |
| Anxiety (GAD-7≥5 score) ^b^ | 9 (4.92%) |

BMI: body mass index; DM: diabetes; WBC, white blood cell; Hb: hemoglobin; PLT: platellet; UA: Uric acid; TC: total cholesterol; TG: triglyceride; TB: total bilirubin; DB: direct bilirubin; HDL: high density lipoprotein; LDL: low density lipoprotein; ALT: alanine aminotransferase; AST: aspartate aminotransferase; CRP: C-reactive protein; NIHSS: National Institutes of Health Stroke Scale; BI: Barthel Index; PHQ-9: patient health questionnaire-9; GAD-7: generalized anxiety disorder-7.

**Supplementary results of the sensitivity analysis by excluding participants with missing predictors**

**Table S2.** **Comparison of the baseline characteristics of elderly stroke participants between good sleep quality and poor sleep quality groups in the sensitivity analysis by excluding participants with missing predictors**

|  | **PSQI≤7 (n=91)** | **PSQI****>7(n=67)** | ***P*** |
| --- | --- | --- | --- |
| Gender, female ^b^ | 56 (61.50) | 43 (64.20) | 0.735 |
| Age (years) ^c^ | 73.00 (67.50, 78.50) | 76.00 (68.50, 83.50) | 0.053 |
| BMI (kg/m^2^) ^a^ | 24.45±3.47 | 23.79±4.51 | 0.319 |
| Residence^b^ |  |  | 0.972 |
| City | 41 (45.10) | 30 (44.80) |  |
| Country | 50 (54.90) | 37 (55.20) |  |
| Marital status ^b^ |  |  | 0.175 |
| Married | 71 (78.00) | 43 (64.20) |  |
| Unmarried | 2 (2.20) | 1 (1.50) |  |
| Widowed | 16 (17.60) | 22 (32.80) |  |
| Divorced | 2 (2.20) | 1 (1.50) |  |
| Education ^b^ |  |  | 0.661 |
| Primary school and below | 31 (34.10) | 18 (26.90) |  |
| Junior high school or technical secondary school | 24 (26.40) | 23 (34.30) |  |
| High school or junior college | 21 (23.10) | 14 (20.90) |  |
| Bachelor degree or above | 15 (16.50) | 12 (17.90) |  |
| Income/year (RMB) ^b^ |  |  | 0.214 |
| ＜15000 | 40 (44.00) | 21 (31.30) |  |
| 15000-33000 | 28 (30.80) | 22 (32.80) |  |
| ＞33000 | 23 (25.30) | 24 (35.80) |  |
| Medical insurance, yes ^b^ | 76 (83.50) | 56 (83.60) | 0.838 |
| Current Drinking, yes ^b^ | 16 (17.60) | 19 (28.40) | 0.107 |
| Current smoking, yes ^b^ | 17 (18.70) | 25 (37.30) | ***0.011*** |
| Stroke history, yes ^b^ | 11 (12.10) | 18 (26.90) | ***0.018*** |
| Comorbidities ^b^ |  |  |  |
| Hypertension | 45 (49.50) | 48 (71.60) | ***0.008*** |
| DM | 37 (40.70) | 27 (40.30) | 0.964 |
| Hyperlipidemia | 45 (49.50) | 31 (46.30) | 0.748 |
| Medications use ^b^ |  |  |  |
| Antiplatelet agents | 3 (3.30) | 2 (3.00) | 0.912 |
| Anticoagulant | 0 (0.00) | 1 (1.50) | 0.424 |
| Lipid-lowering agents | 2 (2.20) | 2 (3.00) | 0.756 |
| Lmphocyte count (10^9^/L) ^c^ | 1.34 (1.04, 1.77) | 1.17 (0.83, 1.44) | 0.211 |
| Neutrophil count (10^9^/L) ^c^ | 4.90 (3.85, 6.91) | 5.10 (3.76, 7.27) | 0.137 |
| WBC count (10^9^/L) ^c^ | 7.06 (5.55, 9.23) | 7.05 (5.52, 9.45) | 0.425 |
| Hb (g/L) ^a^ | 128.75±22.03 | 116.03±24.82 | ***0.001*** |
| UA (µmol/L) ^c^ | 347.50 (297.00, 394.00) | 426.50 (286.00, 538.00) | ***0.001*** |
|  | **PSQI≤7 (n=91)** | **PSQI>7(n=67)** | ***P*** |
| PLT (10^9^/L) ^c^ | 184.50 (151.00, 222.00) | 170.00 (137.00, 231.00) | 0.855 |
| TC (µmol/L) ^c^ | 4.03 (3.36, 4.68) | 3.30 (2.83, 4.19) | 0.172 |
| TG (µmol/L) ^c^ | 1.25 (0.97, 1.92) | 1.21 (0.86, 1.61) | 0.750 |
| TB (µmol/L) ^c^ | 12.89 (10.48, 19.10) | 12.66 (9.42, 17.30) | 0.451 |
| DB (µmol/L) ^c^ | 2.52 (1.66, 3.72) | 2.47 (1.61, 5.57) | 0.242 |
| LDL (mmol/L) ^c^ | 2.39 (1.87, 2.83) | 2.05 (1.69, 2.87) | 0.416 |
| HDL (mmol/L) ^c^ | 0.98 (0.84, 1.20) | 0.99 (0.75, 1.16) | 0.290 |
| ALT (IU/L) ^c^ | 23.00 (16.00, 41.00) | 18.00 (12.00, 34.00) | 0.520 |
| AST (IU/L) ^a^ | 27.00 (20.00, 60.44) | 26.00 (15.00, 54.00) | 0.424 |
| Serum albumin (mmol/L) ^c^ | 139.25 (137.30, 141.10) | 139.25 (136.80, 141.40) | 0.665 |
| Serum potassium (mmol/L) ^c^ | 4.00 (3.80, 4.20) | 4.20 (3.80, 4.50) | 0.419 |
| Serum calcium (mmol/L) ^c^ | 2.24 (2.17, 2.29) | 2.20 (2.09, 2.30) | 0.531 |
| CRP (mg/L) ^c^ | 4.27 (1.49, 12.38) | 8.90 (3.01, 21.76) | 0.328 |
| Hcrt-1 (pg/ml) | 51.91 (41.91, 63.67) | 70.79(54.82, 82.98) | ***<0.001*** |
| NIHSS score ^c^ | 3.5 (3, 4) | 4 (3, 5) | ***0.001*** |
| BI ^a^ | 61.39±21.34 | 57.41±21.27 | 0.098 |
| Mechanical ventilation ^b^ | 7 (7.70) | 7 (10.40) | 0.581 |
| Physical restraint ^b^ | 13 (14.30) | 16 (23.90) | 0.147 |
| Gastric tube ^b^ | 2 (2.20) | 6 (9.00) | 0.072 |
| Indwelling catheter ^b^ | 16 (17.60) | 17 (25.40) | 0.243 |
| Depression (PHQ-9≥5 score) ^b^ | 16 (17.60) | 39 (58.20) | ***<0.001*** |
| Anxiety (GAD-7≥5 score) ^b^ | 9 (9.90) | 18 (26.80) | ***0.005*** |

PSQI: Pittsburgh Sleep Quality Index.

**Table S3. LRs, AICs, BICs, and AUCs of Model 1 and Model 2 in the sensitivity analysis by excluding participants with missing predictors (Hcrt-1 as continuous variable)**

|  | **Likelihood tests** | | **AIC** | **BIC** | **ROC tests** | | | |
| --- | --- | --- | --- | --- | --- | --- | --- | --- |
|  | **LR** | ***P*** |  |  | **AUC of Model 1** | **AUC of Model 2** | ***Z*** | ***P*** |
| **Prior to bootstrapping by excluding participants with missing predictors** | 33.143 | ***<0.001*** | 173.936 | 189.158 | 0.784  (0.728, 0.823) | 0.835  (0.789, 0.902) | 2.035 | ***0.015*** |
| **After bootstrapping internal validation by excluding participants with missing predictors** | 31.330 | ***<0.001*** | 158.264 | 176.564 | 0.809  (0.745, 0.873) | 0.860  (0.805, 0.915) | 2.194 | ***0.028*** |

Likelihood Ratio

LR: Likelihood Ratio; AIC, Akaike's Information Criterion; BIC: Bayesian Information Criterion; ROC, Receiver Operating Characteristic; AUC, area under receiver operating characteristics curve.

**Table S4. LRs, AICs, BICs, and AUCs of Model 1 and Model 2 in the sensitivity analysis by excluding participants with missing predictors (Hcrt-1 as binary variable)**

|  | **Likelihood tests** | | **AIC** | **BIC** | **ROC tests** | | | |
| --- | --- | --- | --- | --- | --- | --- | --- | --- |
|  | **LR** | ***P*** |  |  | **AUC of Model 1** | **AUC of Model 2** | ***Z*** | ***P*** |
| **Prior to bootstrapping by excluding participants with missing predictors** | 35.921 | ***<0.001*** | 165.163 | 167.125 | 0.768  (0.708, 0.845) | 0.848  (0.768, 0.921) | 2.623 | ***0.013*** |
| **After bootstrapping internal validation by excluding participants with missing predictors** | 32.356 | ***<0.001*** | 146.717 | 165.012 | 0.721  (0.654, 0.804) | 0.803  (0.743, 0.875) | 2.417 | ***0.021*** |

LR: Likelihood Ratio; AIC, Akaike's Information Criterion; BIC: Bayesian Information Criterion; ROC, Receiver Operating Characteristic; AUC, area under receiver operating characteristics curve.

**Fig S1. Bias-corrected calibration plots of Model 1 and Model 2 for after bootstrapping in the sensitivity analysis by excluding participants with missing predictors (Hcrt-1 as continuous variable)**

**
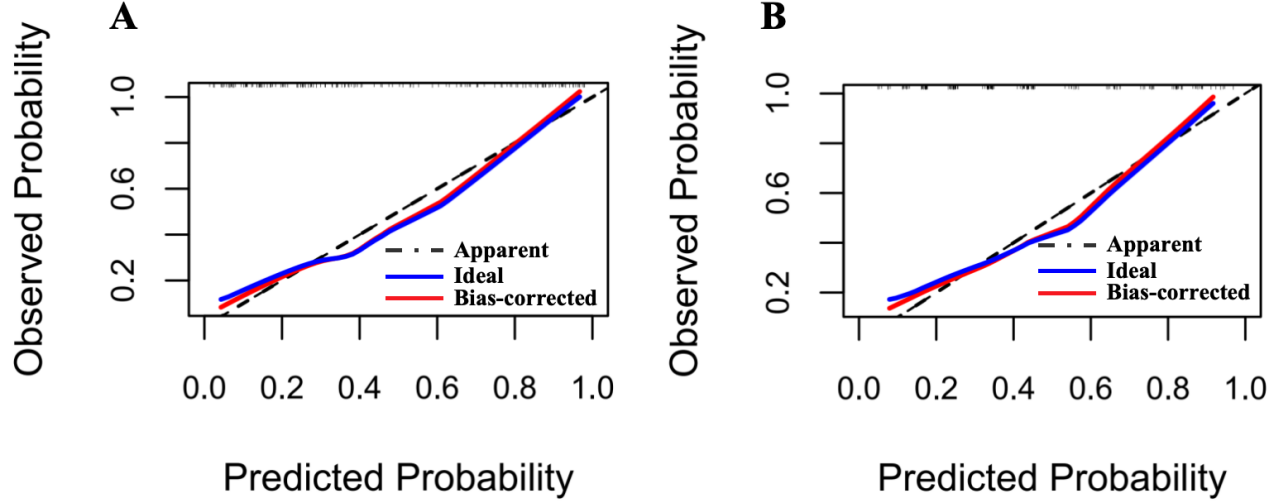
**

(A) Calibration plots of Model 1 after bootstrapping in the sensitivity analysis by excluding participants with missing predictors. (B) Calibration plots of Model 2 after bootstrapping in the sensitivity analysis by excluding participants with missing predictors.

**Fig S2. Bias-corrected calibration plots of Model 1 and Model 2 for after bootstrapping in the sensitivity analysis by excluding participants with missing predictors (Hcrt-1 as binary variable)**

**
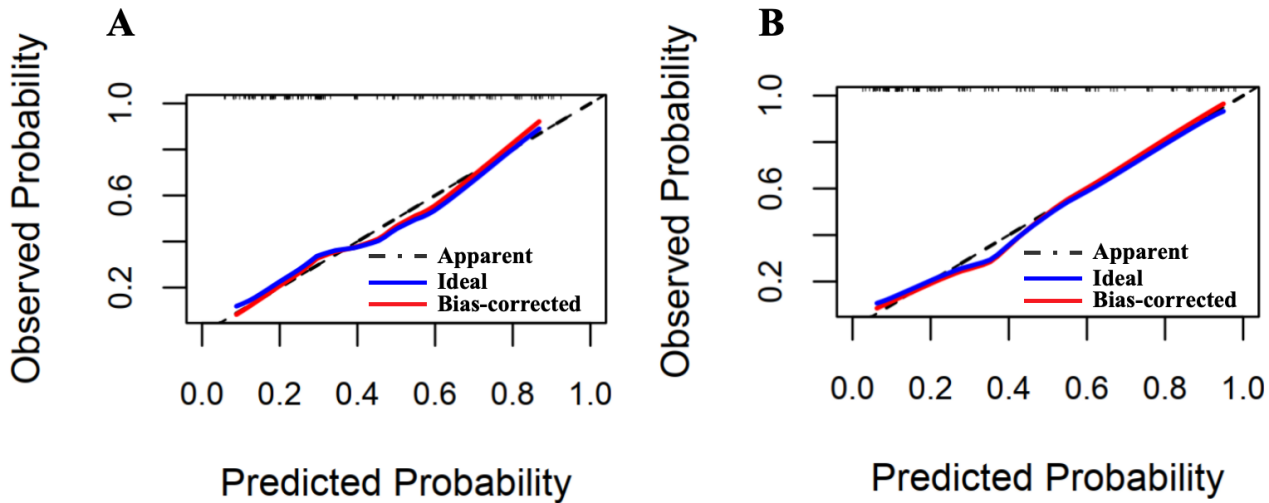
**

(A) Calibration plots of Model 1 after bootstrapping in the sensitivity analysis by excluding participants with missing predictors. (B) Calibration plots of Model 2 after bootstrapping in the sensitivity analysis by excluding participants with missing predictors.

**Figure S3. Bias-corrected decision curve analysis of Model 1 and Model 2 after bootstrapping in the sensitivity analysis by excluding participants with missing predictors**


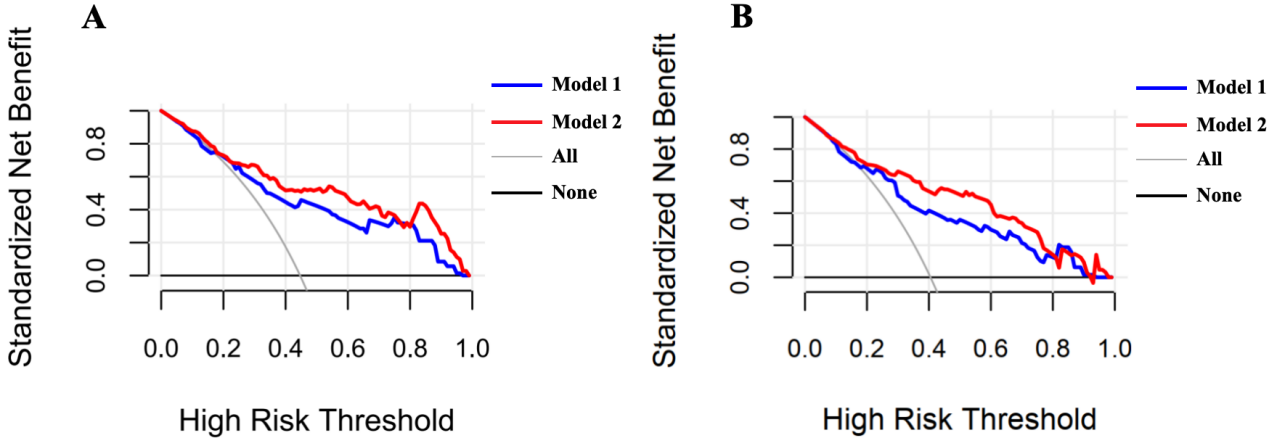


(A) Bias-corrected decision curve analysis of Model 1 and Model 2 after bootstrapping in the sensitivity analysis by excluding participants with missing predictors (Hcrt-1 as continuous variable). (B) Bias-corrected decision curve analysis of Model 1 and Model 2 after bootstrapping in the sensitivity analysis by excluding participants with missing predictors (Hcrt-1 as binary variable).

**Supplementary results of the sensitivity analysis by excluding participants using mechanical ventilation**

**Table S5. Comparison of the baseline characteristics of elderly stroke participants between good sleep quality and poor sleep quality groups in the sensitivity analysis by excluding participants with mechanical ventilation**

|  | **PSQI≤7 (n=98)** | **PSQI>7(n=71)** | | ***P*** |
| --- | --- | --- | --- | --- |
| Gender, female ^b^ | 60 (61.20) | 48 (67.60) | | 0.421 |
| Age (years) ^c^ | 73.00 (67.00, 79.00) | 75.00 (67.00, 82.00) | | 0.298 |
| BMI (kg/m^2^) ^a^ | 24.43±3.30 | 23.60±4.06 | | 0.159 |
| Residence^b^ |  |  | | 0.875 |
| City | 42 (42.90) | 32 (45.10) | |  |
| Country | 56 (57.10) | 39 (54.90) | |  |
| Marital status ^b^ |  |  | | 0.175 |
| Married | 77 (78.60) | 48 (67.60) | |  |
| Unmarried | 2 (2.00) | 2 (2.80) | |  |
| Widowed | 17 (17.30) | 20 (28.20) | |  |
| Divorced | 2 (2.00) | 1 (1.40) | |  |
| Education ^b^ |  |  | | 0.381 |
| Primary school and below | 25 (25.50) | 19 (26.80) | |  |
| Junior high school or technical secondary school | 32 (32.70) | 27 (38.00) | |  |
| High school or junior college | 24 (24.50) | 14 (19.70) | |  |
| Bachelor degree or above | 17 (17.30) | 11 (15.50) | |  |
| Income/year (RMB) ^b^ |  |  | | 0.860 |
| ＜15000 | 37 (37.80) | 28 (39.40) | |  |
| 15000-33000 | 31 (31.60) | 24 (33.80) | |  |
| ＞33000 | 30 (30.60) | 19 (26.80) | |  |
| Medical insurance, yes ^b^ | 78 (79.60) | 60 (84.50) | | 0.223 |
| Current Drinking, yes ^b^ | 21 (21.40) | 19 (26.80) | | 0.466 |
| Current smoking, yes ^b^ | 18 (18.40) | 22 (31.00) | | 0.068 |
| Stroke history, yes ^b^ | 12 (12.20) | 17 (23.00) | | ***0.046*** |
| Comorbidities ^b^ |  |  | |  |
| Hypertension | 53 (54.10) | 49 (69.00) | | ***0.050*** |
| DM | 37 (37.80) | 29 (40.80) | | 0.750 |
| Hyperlipidemia | 49 (50.00) | 34 (47.90) | | 0.876 |
| Medications use ^b^ |  |  | |  |
| Antiplatelet agents | 6 (6.10) | 3 (4.20) | | 0.736 |
| Anticoagulant | 0 (0.00) | 1 (1.40) | | 0.420 |
| Lipid-lowering agents | 0 (0.00) | 1 (1.40) | | 0.420 |
| Lmphocyte count (10^9^/L) ^c^ | 1.34 (1.04, 1.81) | 1.28 (0.81, 1.54) | | 0.247 |
| Neutrophil count (10^9^/L) ^c^ | 4.92 (3.85, 6.78) | 4.93 (3.48, 6.72) | | 0.333 |
| WBC count (10^9^/L) ^c^ | 7.12 (5.55, 9.35) | 7.05 (5.08, 9.33) | | 0.510 |
| Hb (g/L) ^a^ | 129.84±19.99 | 117.79±25.92 | | ***0.001*** |
|  | **PSQI≤7 (n=98)** | **PSQI>7(n=71)** | | ***P*** |
| UA (µmol/L) ^c^ | 350.00 (297.00, 394.00) | 396.50 (285.00, 498.00) | | 0.091 |
| PLT (10^9^/L) ^c^ | 188.00 (162.00, 233.00) | 173.50 (152.00, 239.00) | 0.626 | |
| TC (µmol/L) ^c^ | 3.96 (3.36, 4.68) | 3.45 (2.83, 4.26) | | 0.405 |
| TG (µmol/L) ^c^ | 1.25 (0.93, 2.07) | 1.22 (0.84, 1.64) | | 0.393 |
| TB (µmol/L) ^c^ | 13.13 (11.03, 18.43) | 13.15 (9.42, 16.45) | | 0.425 |
| DB (µmol/L) ^c^ | 2.59 (1.74, 3.94) | 2.47 (1.74, 3.99) | | 0.184 |
| LDL (mmol/L) ^c^ | 2.37 (1.86, 2.78) | 2.08 (1.68, 2.72) | | 0.829 |
| HDL (mmol/L) ^c^ | 1.02 (0.89, 1.23) | 1.00 (0.82, 1.20) | | 0.323 |
| ALT (IU/L) ^c^ | 23.00 (15.00, 38.00) | 19.50 (13.00, 38.00) | | 0.083 |
| AST (IU/L) ^a^ | 26.00 (19.00, 53.00) | 25.50 (17.00, 55.00) | | 0.739 |
| Serum albumin (mmol/L) ^c^ | 139.20 (137.00, 140.70) | 139.55 (137.20, 141.40) | | 0.840 |
| Serum potassium (mmol/L) ^c^ | 4.00 (3.80, 4.20) | 4.20 (3.80, 4.50) | | 0.418 |
| Serum calcium (mmol/L) ^c^ | 2.24 (2.19, 2.30) | 2.21 (2.11, 2.31) | | 0.531 |
| CRP (mg/L) ^c^ | 3.68 (1.39, 11.09) | 7.10 (2.50, 17.92) | | 0.489 |
| Hcrt-1 (pg/ml) | 51.35 (40.79, 63.19) | 70.34(54.64, 82.06) | | ***<0.001*** |
| NIHSS score ^c^ | 3.0 (3, 4) | 4 (4, 5) | | ***<0.001*** |
| BI ^a^ | 63.61±22.53 | 58.23±21.25 | | 0.120 |
| Physical restraint ^b^ | 12 (12.20) | 13 (18.30) | | 0.147 |
| Gastric tube ^b^ | 1 (1.00) | 5 (7.00) | | 0.240 |
| Indwelling catheter ^b^ | 12 (12.20) | 14 (19.70) | | 0.201 |
| Depression (PHQ-9≥5 score) ^b^ | 18 (18.40) | 42 (59.20) | | ***<0.001*** |
| Anxiety (GAD-7≥5 score) ^b^ | 11 (11.20) | 21 (29.60) | | ***0.005*** |

**Table S6. LRs, AICs, BICs, and AUCs of Model 1 and Model 2 in the sensitivity analysis by excluding participants with mechanical ventilation (Hcrt-1 as continuous variable)**

|  | **Likelihood tests** | | **AIC** | **BIC** | **ROC tests** | | | |
| --- | --- | --- | --- | --- | --- | --- | --- | --- |
|  | **LR** | ***P*** |  |  | **AUC of Model 1** | **AUC of Model 2** | ***Z*** | ***P*** |
| **Prior to bootstrapping by excluding participants with mechanical ventilation** | 34.431 | ***<0.001*** | 188.777 | 204.426 | 0.808  (0.741, 0.875) | 0.865  (0.808, 0.922) | 2.252 | ***0.024*** |
| **After bootstrapping internal validation by excluding participants with mechanical ventilation** | 31.192 | ***<0.001*** | 158.559 | 177.338 | 0.788  (0.716, 0.860) | 0.851  (0.791, 0.910) | 2.370 | ***0.038*** |

LR: Likelihood Ratio; AIC, Akaike's Information Criterion; BIC: Bayesian Information Criterion; ROC, Receiver Operating Characteristic; AUC, area under receiver operating characteristics curve.

**Table S7. LRs, AICs, BICs, and AUCs of Model 1 and Model 2 in the sensitivity analysis by excluding participants with mechanical ventilation (Hcrt-1 as binary variable)**

|  | **Likelihood tests** | | **AIC** | **BIC** | **ROC tests** | | | |
| --- | --- | --- | --- | --- | --- | --- | --- | --- |
|  | **LR** | ***P*** |  |  | **AUC of Model 1** | **AUC of Model 2** | ***Z*** | ***P*** |
| **Prior to bootstrapping by excluding participants with mechanical ventilation** | 33.925 | ***<0.001*** | 168.325 | 201.013 | 0.811  (0.753, 0.879) | 0.871  (0.814, 0.916) | 2.257 | ***0.021*** |
| **After bootstrapping internal validation by excluding participants with mechanical ventilation** | 30.962 | ***<0.001*** | 160.829 | 179.608 | 0.793  (0.723, 0.865) | 0.858  (0.797, 0.919) | 2.376 | ***0.027*** |

LR: Likelihood Ratio; AIC, Akaike's Information Criterion; BIC: Bayesian Information Criterion; ROC, Receiver Operating Characteristic; AUC, area under receiver operating characteristics curve.

**Fig S4. Bias-corrected calibration plots of Model 1 and Model 2 for after bootstrapping in the sensitivity analysis by excluding participants with mechanical ventilation (Hcrt-1 as continuous variable)**


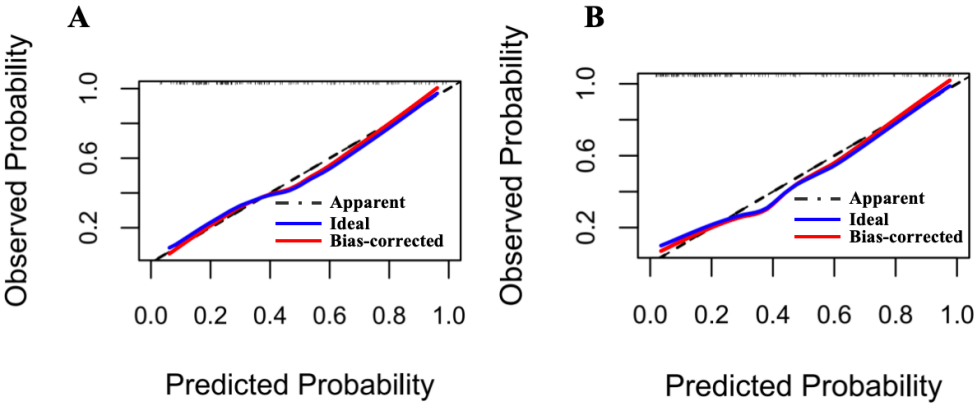


(A) Calibration plots of Model 1 after bootstrapping in the sensitivity analysis by excluding participants with mechanical ventilation. (B) Calibration plots of Model 2 after bootstrapping in the sensitivity analysis by excluding participants with mechanical ventilation.

**Fig S5. Bias-corrected calibration plots of Model 1 and Model 2 for after bootstrapping in the sensitivity analysis by excluding participants with mechanical ventilation (Hcrt-1 as binary variable)**

**
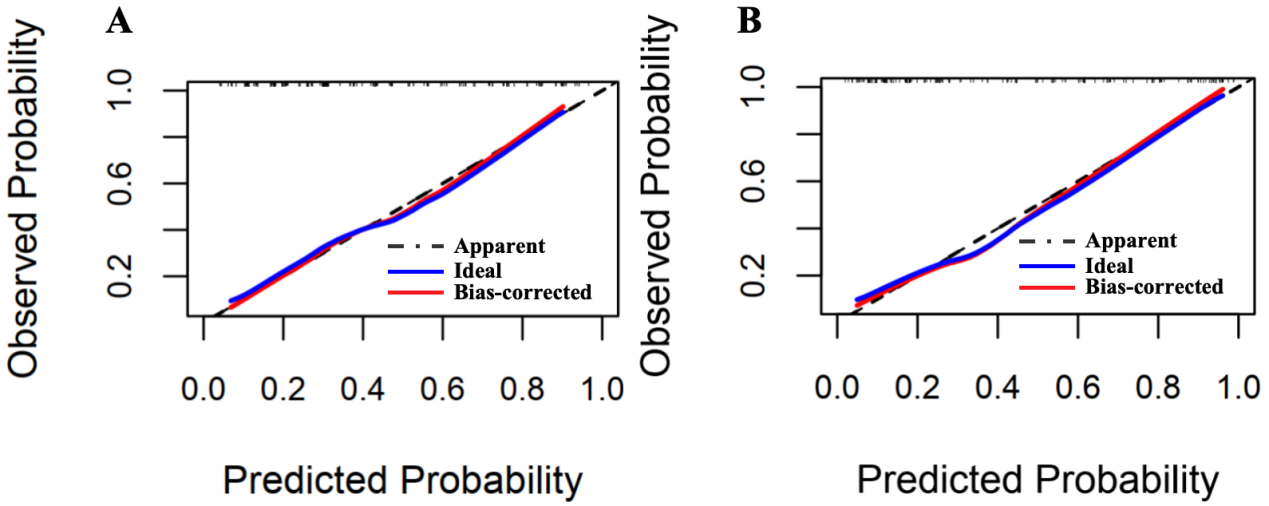
**

(A) Calibration plots of Model 1 after bootstrapping in the sensitivity analysis by excluding participants with mechanical ventilation. (B) Calibration plots of Model 2 after bootstrapping in the sensitivity analysis by excluding participants with mechanical ventilation.

**Figure S6. Bias-corrected decision curve analysis of Model 1 and Model 2 after bootstrapping in the sensitivity analysis by excluding participants with mechanical ventilation**

**
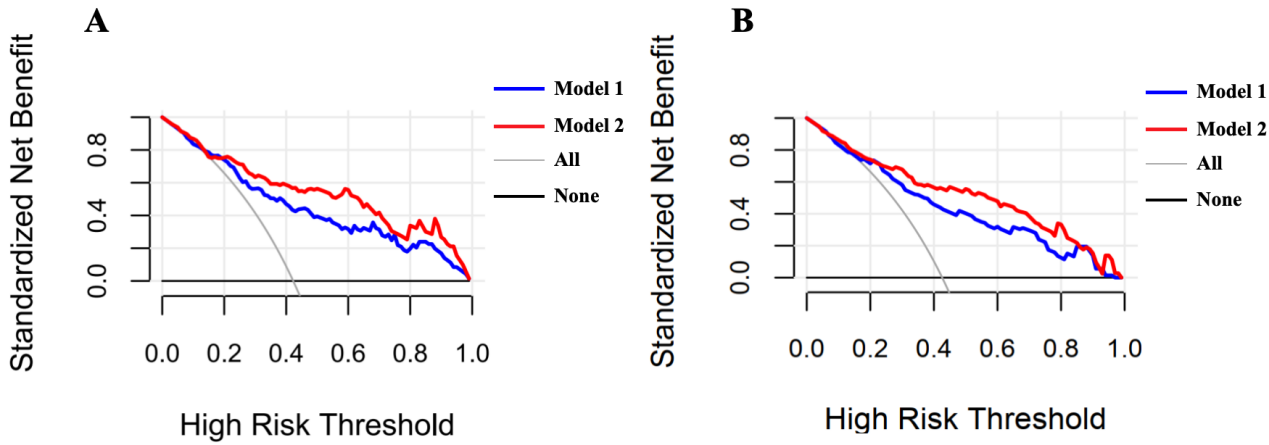
**

(A) Bias-corrected decision curve analysis of Model 1 and Model 2 after bootstrapping in the sensitivity analysis by excluding participants with missing predictors (Hcrt-1 as continuous variable). (B) Bias-corrected decision curve analysis of Model 1 and Model 2 after bootstrapping in the sensitivity analysis by excluding participants with missing predictors (Hcrt-1 as binary variable).

**Supplementary results of the sensitivity analysis by excluding participants using physical restraint**

**Table S8. Comparison of the baseline characteristics of elderly stroke participants between good sleep quality and poor sleep quality groups in the sensitivity analysis by excluding participants with physical restraint**

|  | **PSQI≤7 (n=92)** | **PSQI>7(n=62)** | ***P*** |
| --- | --- | --- | --- |
| Gender, female ^b^ | 56 (60.90) | 42 (67.70) | 0.399 |
| Age (years) ^c^ | 73.00 (65.00, 79.00) | 76.00 (66.00, 81.00) | 0.652 |
| BMI (kg/m^2^) ^a^ | 24.56±3.34 | 24.15±4.31 | 0.532 |
| Residence^b^ |  |  | 0.287 |
| City | 38 (41.30) | 31 (50.00) |  |
| Country | 54 (58.70) | 31 (50.00) |  |
| Marital status ^b^ |  |  | 0.253 |
| Married | 73 (79.30) | 41 (66.10) |  |
| Unmarried | 1 (1.10) | 2 (3.20) |  |
| Widowed | 16 (17.40) | 18 (29.00) |  |
| Divorced | 2 (2.20) | 1 (1.60) |  |
| Education ^b^ |  |  | 0.324 |
| Primary school and below | 31 (33.70) | 20 (32.30) |  |
| Junior high school or technical secondary school | 23 (25.00) | 23 (37.10) |  |
| High school or junior college | 24 (26.10) | 10 (16.10) |  |
| Bachelor degree or above | 14 (15.20) | 9 (17.90) |  |
| Income/year (RMB) ^b^ |  |  | 0.928 |
| ＜15000 | 31 (34.10) | 23 (37.10) |  |
| 15000-33000 | 34 (37.40) | 22 (35.50) |  |
| ＞33000 | 26 (28.60) | 27 (27.40) |  |
| Medical insurance, yes ^b^ | 76 (83.50) | 60 (96.80) | 0.838 |
| Current Drinking, yes ^b^ | 20 (21.70) | 16 (25.80) | 0.559 |
| Current smoking, yes ^b^ | 17 (18.50) | 20 (32.30) | 0.056 |
| Stroke history, yes ^b^ | 11 (12.10) | 18 (26.90) | 0.052 |
| Comorbidities ^b^ |  |  |  |
| Hypertension | 45 (49.50) | 48 (71.60) | ***0.008*** |
| DM | 37 (40.70) | 27 (40.30) | 0.964 |
| Hyperlipidemia | 45 (49.50) | 31 (46.30) | 0.748 |
| Medications use ^b^ |  |  |  |
| Antiplatelet agents | 3 (3.30) | 2 (3.00) | 0.912 |
| Anticoagulant | 0 (0.00) | 1 (1.50) | 0.424 |
| Lipid-lowering agents | 2 (2.20) | 2 (3.00) | 0.756 |
| Lmphocyte count (10^9^/L) ^c^ | 1.39 (1.05, 1.82) | 1.28 (0.83, 1.54) | 0.103 |
| Neutrophil count (10^9^/L) ^c^ | 4.89 (3.82, 6.67) | 4.75 (3.48, 6.25) | 0.333 |
| WBC count (10^9^/L) ^c^ | 7.03 (5.44, 9.05) | 6.64 (5.19, 8.59) | 0.635 |
| UA (µmol/L) ^c^ | 350.00 (297.00, 395.00) | 401.00 (287.00, 518.00) | 0.091 |
| Hb (g/L) ^a^ | 133.00 (122.00, 140.50) | 117.00 (98.00, 135.00) | 0.210 |
|  | **PSQI≤7 (n=92)** | **PSQI>7(n=62)** | ***P*** |
| PLT (10^9^/L) ^c^ | 189.00 (164.50, 232.00) | 170.00 (152.00, 231.00) | 0.265 |
| TC (µmol/L) ^c^ | 3.97 (3.37, 4.71) | 3.53 (2.83, 4.26) | 0.440 |
| TG (µmol/L) ^c^ | 1.25 (0.96, 2.09) | 1.23 (0.84, 1.71) | 0.494 |
| TB (µmol/L) ^c^ | 12.98 (10.888, 18.27) | 11.97 (8.49, 16.47) | 0.425 |
| DB (µmol/L) ^c^ | 2.53 (1.70, 3.78) | 2.24 (1.61, 3.52) | 0.277 |
| LDL (mmol/L) ^c^ | 2.40 (1.88, 2.83) | 2.11 (1.69, 2.79) | 0.337 |
| HDL (mmol/L) ^c^ | 0.98 (0.89, 1.23) | 1.00 (0.85, 1.16) | 0.381 |
| ALT (IU/L) ^c^ | 23.00 (15.50, 37.50) | 19.00 (12.00, 33.00) | 0.249 |
| AST (IU/L) ^a^ | 26.00 (19.50, 48.50) | 24.00 (15.00, 54.00) | 0.332 |
| Serum albumin (mmol/L) ^c^ | 139.20 (137.30, 140.80) | 138.70 (136.80, 140.80) | 0.229 |
| Serum potassium (mmol/L) ^c^ | 4.00 (3.80, 4.20) | 4.20 (3.80, 4.20) | 0.438 |
| Serum calcium (mmol/L) ^c^ | 2.24 (2.19, 2.30) | 2.20 (2.11, 2.29) | 0.051 |
| CRP (mg/L) ^c^ | 3.41 (1.27, 8.92) | 5.76 (2.22, 15.33) | 0.069 |
| Hcrt-1 (pg/ml) | 49.68 (40.77, 62.19) | 68.44(54.63, 82.06) | ***<0.001*** |
| NIHSS score ^c^ | 3.5 (3, 4) | 4 (3, 5) | ***0.001*** |
| BI ^a^ | 64.13±22.59 | 57.42±21.23 | 0.066 |
| Mechanical ventilation ^b^ | 7 (7.70) | 7 (10.40) | 0.581 |
| Gastric tube ^b^ | 2 (2.20) | 6 (9.00) | 0.072 |
| Indwelling catheter ^b^ | 16 (17.60) | 17 (25.40) | 0.243 |
| Depression (PHQ-9≥5 score) ^b^ | 17 (18.50) | 35 (56.50) | ***<0.001*** |
| Anxiety (GAD-7≥5 score) ^b^ | 10 (10.90) | 18 (29.00) | ***0.006*** |

**Table S9. LRs, AICs, BICs, and AUCs of Model 1 and Model 2 in the sensitivity analysis by excluding participants with physical restraint (Hcrt-1 as continuous variable)**

|  | **Likelihood tests** | | **AIC** | **BIC** | **ROC tests** | | | |
| --- | --- | --- | --- | --- | --- | --- | --- | --- |
|  | **LR** | ***P*** |  |  | **AUC of Model 1** | **AUC of Model 2** | ***Z*** | ***P*** |
| **Prior to bootstrapping by excluding participants with physical restraint** | 30.925 | ***<0.001*** | 219.891 | 235.076 | 0.792  (0.718, 0.866) | 0.855  (0.794, 0.917) | 2.458 | ***0.014*** |
| **After bootstrapping internal validation by excluding participants with physical restraint** | 33.463 | ***<0.001*** | 194.108 | 212.330 | 0.788  (0.714, 0.862) | 0.839  (0.775, 0.904) | 2.043 | ***0.041*** |

LR: Likelihood Ratio; AIC, Akaike's Information Criterion; BIC: Bayesian Information Criterion; ROC, Receiver Operating Characteristic; AUC, area under receiver operating characteristics curve.

**Table S10. LRs, AICs, BICs, and AUCs of Model 1 and Model 2 in the sensitivity analysis by excluding participants with physical restraint (Hcrt-1 as binary variable)**

|  | **Likelihood tests** | | **AIC** | **BIC** | **ROC tests** | | | |
| --- | --- | --- | --- | --- | --- | --- | --- | --- |
|  | **LR** | ***P*** |  |  | **AUC of Model 1** | **AUC of Model 2** | ***Z*** | ***P*** |
| **Prior to bootstrapping by excluding participants with physical restraint** | 34.751 | ***<0.001*** | 205.736 | 217.153 | 0.797  (0.723, 0.875) | 0.858  (0.796, 0.921) | 2.466 | ***0.009*** |
| **After bootstrapping internal validation by excluding participants with physical restraint** | 31.742 | ***<0.001*** | 190.960 | 209.182 | 0.794  (0.721, 0.868) | 0.843  (0.781, 0.908) | 2.051 | ***0.035*** |

LR: Likelihood Ratio; AIC, Akaike's Information Criterion; BIC: Bayesian Information Criterion; ROC, Receiver Operating Characteristic; AUC, area under receiver operating characteristics curve.

**Fig S7. Bias-corrected calibration plots of Model 1 and Model 2 for after bootstrapping in the sensitivity analysis by excluding participants with physical restraint (Hcrt-1 as continuous variable)**


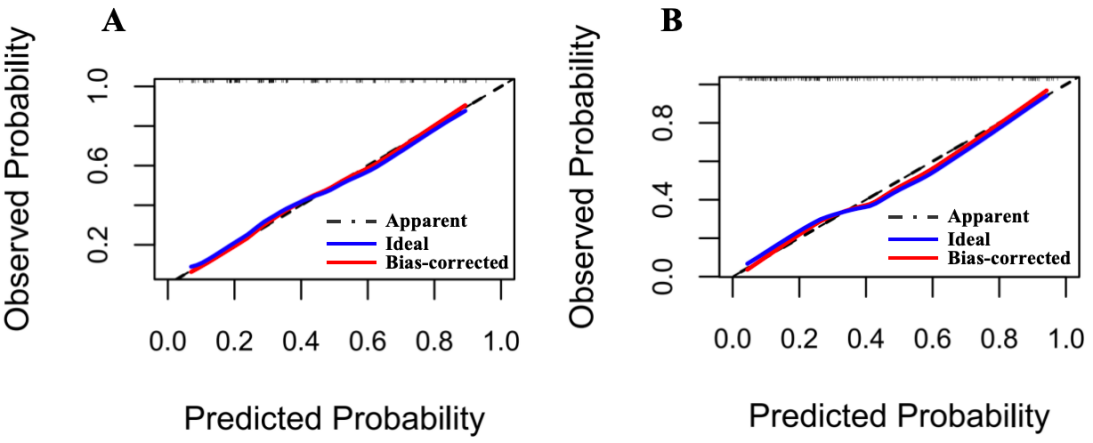


(A) Calibration plots of Model 1 after bootstrapping in the sensitivity analysis by excluding participants with physical restraint. (B) Calibration plots of Model 2 after bootstrapping in the sensitivity analysis by excluding participants with physical restraint.

**Fig S8. Bias-corrected calibration plots of Model 1 and Model 2 for after bootstrapping in the sensitivity analysis by excluding participants with physical restraint (Hcrt-1 as binary variable)**

**
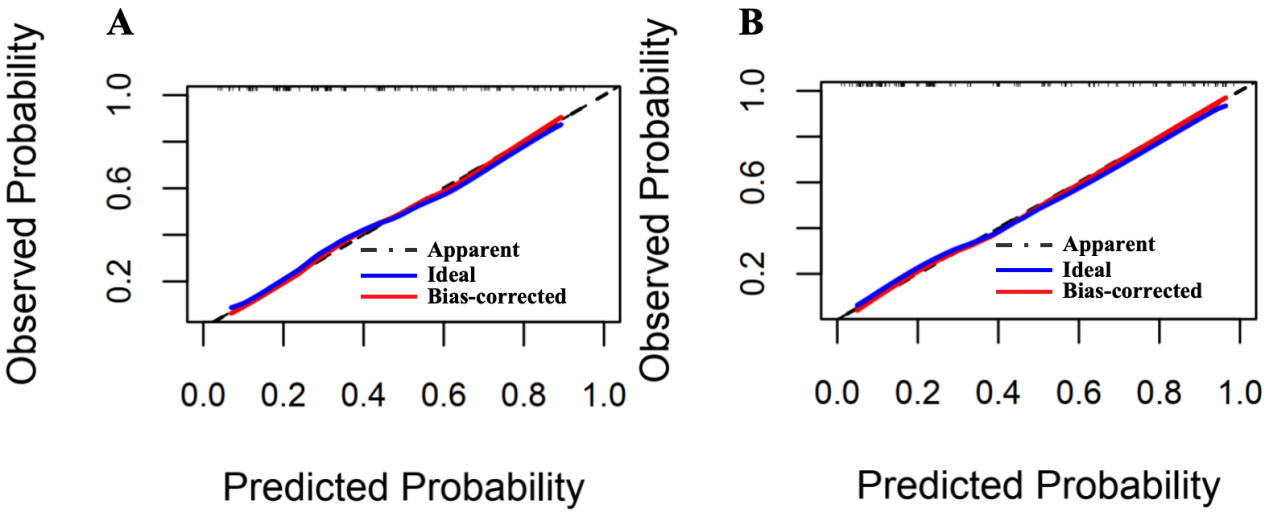
**

(A) Calibration plots of Model 1 after bootstrapping in the sensitivity analysis by excluding participants with physical restraint. (B) Calibration plots of Model 2 after bootstrapping in the sensitivity analysis by excluding participants with physical restraint.

**Figure S9. Bias-corrected decision curve analysis of Model 1 and Model 2 after bootstrapping in the sensitivity analysis by excluding participants with physical restraint**


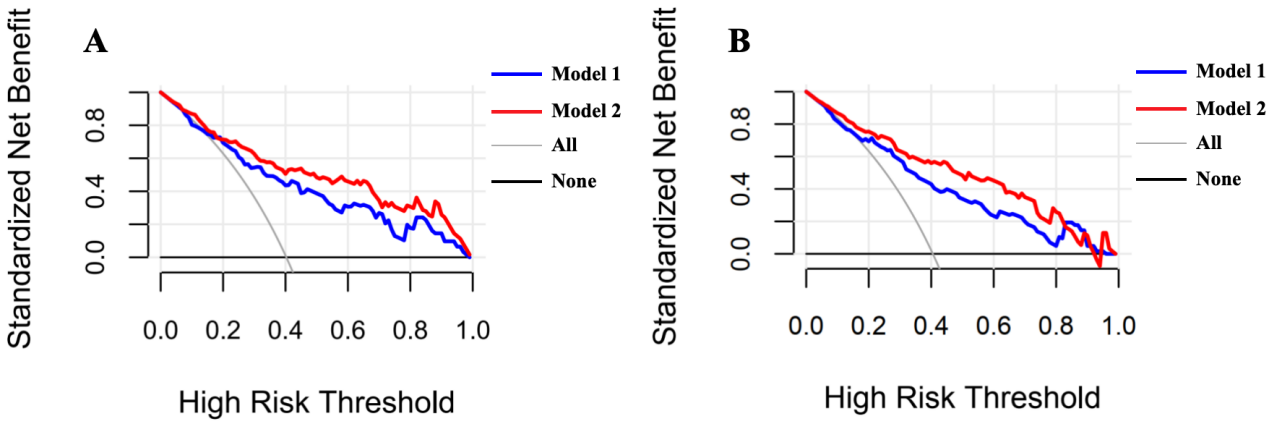


(A) Bias-corrected decision curve analysis of Model 1 and Model 2 after bootstrapping in the sensitivity analysis by excluding participants with missing predictors (Hcrt-1 as continuous variable). (B) Bias-corrected decision curve analysis of Model 1 and Model 2 after bootstrapping in the sensitivity analysis by excluding participants with missing predictors (Hcrt-1 as binary variable).

Supplementary Material should be uploaded separately on submission. Please include any supplementary data, figures and/or tables. Supplementary material is not typeset so please ensure that all information is clearly presented, the appropriate caption is included in the file and not in the manuscript, and that the style conforms to the rest of the article.
